# Effects of ECM protein-coated surfaces on the generation of retinal pigment epithelium cells differentiated from human pluripotent stem cells

**DOI:** 10.1093/rb/rbae091

**Published:** 2024-08-20

**Authors:** Zeyu Tian, Qian Liu, Hui-Yu Lin, Yu-Ru Zhu, Ling Ling, Tzu-Cheng Sung, Ting Wang, Wanqi Li, Min Gao, Sitian Cheng, Remya Rajan Renuka, Suresh Kumar Subbiah, Guoping Fan, Gwo-Jang Wu, Akon Higuchi

**Affiliations:** State Key Laboratory of Ophthalmology, Optometry and Visual Science, Eye Hospital, Wenzhou Medical University, Wenzhou, Zhejiang 325027, China; State Key Laboratory of Ophthalmology, Optometry and Visual Science, Eye Hospital, Wenzhou Medical University, Wenzhou, Zhejiang 325027, China; Department of Chemical and Materials Engineering, National Central University, Taoyuan 32001, Taiwan, China; Department of Chemical and Materials Engineering, National Central University, Taoyuan 32001, Taiwan, China; State Key Laboratory of Ophthalmology, Optometry and Visual Science, Eye Hospital, Wenzhou Medical University, Wenzhou, Zhejiang 325027, China; State Key Laboratory of Ophthalmology, Optometry and Visual Science, Eye Hospital, Wenzhou Medical University, Wenzhou, Zhejiang 325027, China; State Key Laboratory of Ophthalmology, Optometry and Visual Science, Eye Hospital, Wenzhou Medical University, Wenzhou, Zhejiang 325027, China; State Key Laboratory of Ophthalmology, Optometry and Visual Science, Eye Hospital, Wenzhou Medical University, Wenzhou, Zhejiang 325027, China; State Key Laboratory of Ophthalmology, Optometry and Visual Science, Eye Hospital, Wenzhou Medical University, Wenzhou, Zhejiang 325027, China; State Key Laboratory of Ophthalmology, Optometry and Visual Science, Eye Hospital, Wenzhou Medical University, Wenzhou, Zhejiang 325027, China; Center for Global Health Research, Saveetha Medical College and Hospitals, Saveetha Institute of Medical and Technical Sciences, Chennai, Tamil Nadu 602105, India; Center for Global Health Research, Saveetha Medical College and Hospitals, Saveetha Institute of Medical and Technical Sciences, Chennai, Tamil Nadu 602105, India; Department of Human Genetics, David Geffen School of Medicine, UCLA, Los Angeles, CA 90095, USA; Graduate Institute of Medical Sciences and Department of Obstetrics & Gynecology, Tri-Service General Hospital, National Defense Medical Center, Taipei 11490, Taiwan, China; State Key Laboratory of Ophthalmology, Optometry and Visual Science, Eye Hospital, Wenzhou Medical University, Wenzhou, Zhejiang 325027, China; Department of Chemical and Materials Engineering, National Central University, Taoyuan 32001, Taiwan, China; R&D Center for Membrane Technology, Chung Yuan Christian University, Chungli, Taoyuan 320, Taiwan, China

**Keywords:** human pluripotent stem cells, retinal pigment epithelial cells, extracellular matrix, cell differentiation, cell therapy

## Abstract

Retinal degeneration diseases, such as age-related macular degeneration (AMD) and retinitis pigmentosa (RP), initially manifest as dysfunction or death of the retinal pigment epithelium (RPE). Subretinal transplantation of human pluripotent stem cell (hPSC)-derived RPE cells has emerged as a potential therapy for retinal degeneration. However, RPE cells differentiated from hPSCs using current protocols are xeno-containing and are rarely applied in clinical trials. The development of hPSC-derived RPE cell differentiation protocols using xeno-free biomaterials is urgently needed for clinical applications. In this study, two protocols (the activin A and NIC84 protocols) were selected for modification and use in the differentiation of hiPSCs into RPE cells; the chetomin concentration was gradually increased to achieve high differentiation efficiency of RPE cells. The xeno-free extracellular matrix (ECM) proteins, laminin-511, laminin-521 and recombinant vitronectin, were selected as plate-coating substrates, and a Matrigel (xeno-containing ECM)-coated surface was used as a positive control. Healthy, mature hPSC-derived RPE cells were transplanted into 21-day-old Royal College of Surgeons (RCS) rats, a model of retinal degeneration disease. The visual function of RCS rats was evaluated by optomotor response (qOMR) and electroretinography after transplantation of hPSC-derived RPE cells. Our study demonstrated that hPSCs can be efficiently differentiated into RPE cells on LN521-coated dishes using the NIC84 protocol, and that subretinal transplantation of the cell suspensions can delay the progression of vision loss in RCS rats.

## Graphical abstract

**Figure:**
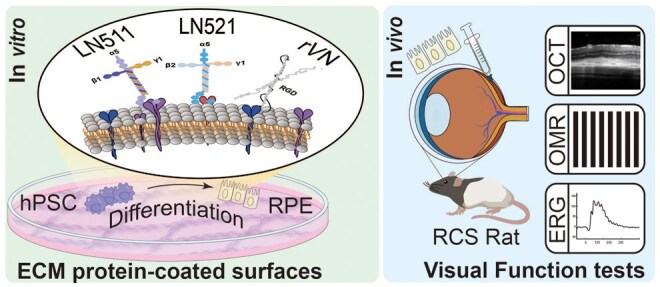

## Introduction

Retinal degeneration (RD) can be caused by various blinding eye diseases, including age-related macular degeneration (AMD), retinitis pigmentosa (RP), hereditary macular atrophy, optical damage, aging, mutations and other conditions [1, 2]. The death or dysfunction of retinal pigment epithelial (RPE) cells and the degeneration or progressive loss of photoreceptor cells are the main causes of blindness in RD [3–5]. The RPE is a highly specialized layer of pigmented cells of neuroectodermal origin that forms between the neurosensory retina and the vascular choroid [6, 7] and it mediates functions that are essential for normal retinal physiology [8].

Stem cell therapies are attracting increasing interest for the treatment of eye injuries and diseases [9–13], although only a few stem cell therapies have been approved [14]. The eye is an attractive site for stem cell applications for several reasons, such as the relatively small number of cells needed, easy accessibility for surgery and ease of visualization of the grafts [15]. Additionally, the eye is considered an immunologically privileged site due to its physical structure and immune system characteristics [16]. As a result, cell therapies for eye diseases have shown promising results in clinical trials, with many types of cells being developed for various blinding disorders, such as corneal disease, cataracts, glaucoma, RP, and AMD [17–20]. The number of native RPE cells is limited, but human pluripotent stem cell (hPSC)-derived RPE cells provide an unlimited source for cell transplantation and are promising therapies; hPSCs is used here to indicate both human embryonic stem cells (hESCs) and human induced pluripotent stem cells (hiPSCs) [21, 22].

The first protocol for generating hESC-derived RPE cells was described in 2004 [23]; however, this protocol is time-consuming and leads to limited *in vivo* survival [24]. To address these issues, researchers have been investigating optimized protocols for rapid, large-scale RPE cell generation for clinical use [25]. Typically, researchers generate RPE cells from hPSCs using a spontaneous differentiation protocol [26, 27], a protocol using small molecules [28–32], or a protocol using neural differentiation factors [21, 33, 34]. Although spontaneous differentiation protocols can efficiently produce mature RPE cells, these protocols are time-consuming, and RPE cells cannot be generated from some hPSC lines [35–37].

RPE differentiation from hPSCs can be accelerated by the addition of several small molecules, such as nicotinamide (NIC) [30], activin A [32], vasoactive intestinal peptide (VIP) [38], SU5402 [21] and chetomin (CTM) [30]. Several studies have used these small molecules to develop protocols for obtaining mature RPE within just 14 days [21, 33]. However, the reproducibility of RPE differentiation from hPSCs via these protocols is unclear, and the generation of RPE cells from hPSCs must occur under completely xeno-free, feeder cell-free and chemically defined conditions to meet the criteria for good manufacturing practices (GMPs) for clinical use [39].

Initially, hPSCs were cultured on mouse embryonic fibroblasts (MEFs) as feeding layers [40, 41]. Matrigel-coated dishes can be used as cell culture biomaterials and can be used in place of MEFs, which are currently the gold standard substrate for hPSC culture [42–44]. However, Matrigel is isolated from an extract of Engelbreth–Holm–Swarm mouse tumors [45], which leads to xenogeneic contamination during cell transplantation therapy [46]. Some extracellular matrix (ECM) proteins not only biochemically support surrounding cells but also interact with cells to regulate multiple functions, including proliferation, migration and differentiation [47–49]. Recombinant human vitronectin (rVN), laminin 511 (LN511) and laminin 521 (LN521) can guide stem cell fate through integrin binding [50]. For example, laminin and laminin-derived peptides can support hPSC differentiation into the neural lineage of cells via α6β1 integrin [51, 52]. Several studies to determine the effects of synthetic substrates and specific protein sequences on stem cell growth and differentiation are underway, and these findings will contribute to the development of new therapies in the future [53–57].

**Figure 1.**
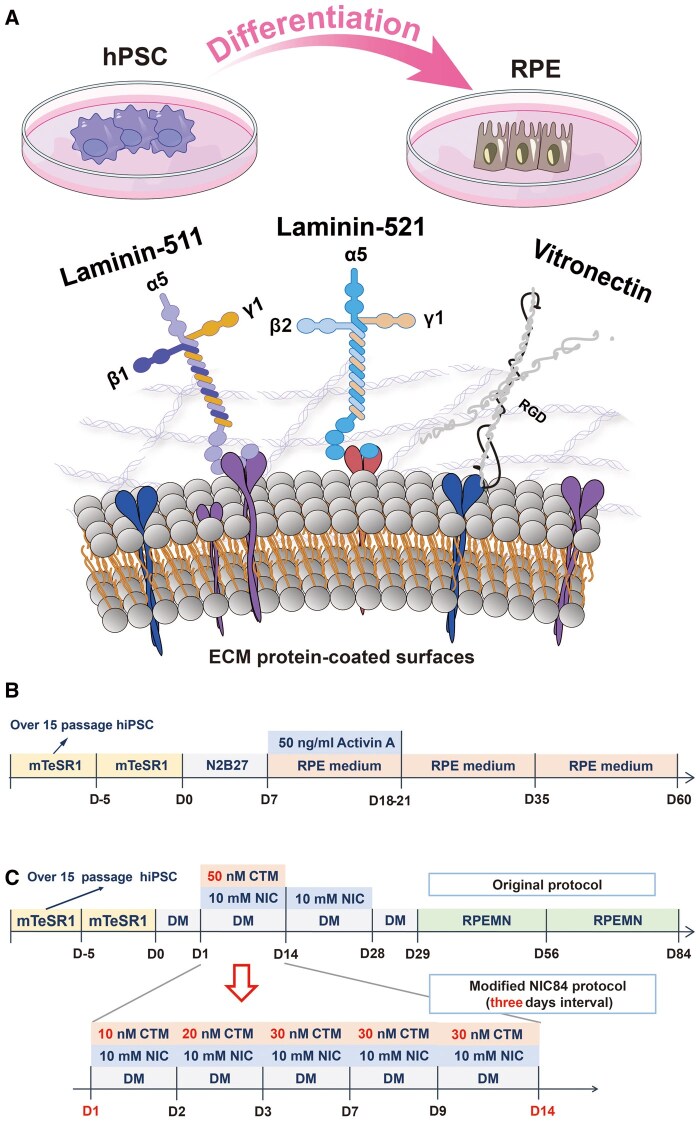
Generation of hPSC-derived RPE cells. (**A**) Illustration of the differentiation of hPSCs into RPE cells on several xeno-free ECM-coated dishes. (**B**) Timeline of the processes for hPSC induction into RPE cells using the activin a protocol. (**C**) Original and modified NIC84 protocols for the induction of hPSCs into RPE cells used in this research.

In this study, efficient differentiation protocols were developed based on two previously reported protocols (the NIC84 [30, 31] and activin A [32] protocols) with modifications (3-day intervals of CTM concentration enhancement), during which four different ECM protein-coated surfaces were evaluated. In general, most researchers have investigated hPSC differentiation into RPE on Matrigel, which is a xeno-containing material [58–60]. Here, we report on the development of an efficient protocol for differentiating hPSCs into RPE cells, which was developed during this study. We also evaluated four different ECM protein-coated surfaces (Matrigel, rVN, LN-511 and LN-521) for the efficient differentiation of hPSCs into RPE cells on xeno-free cell culture materials (Figure 1A).

## Materials and methods

### Preparation of ECM protein-coated dishes

ECM protein-coated dishes were prepared through the following procedure. First, four coating solutions (Matrigel, rVN, LN511 and LN521) were prepared, and Matrigel (356230, Corning, Bedford, USA) was applied as suggested by the manufacturer (10 mg/ml). rVN (A14700, Gibco, Frederick, USA) was diluted to 5 mg/ml with Dulbecco’s phosphate-buffered saline (DPBS) without calcium or magnesium (14190144, Gibco, Grand Island, USA). LN511 (LN511-0202; BioLamina, Sundbyberg, Sweden) or LN521 (LN521-02; BioLamina, Sundbyberg, Sweden) was diluted to 5 mg/ml with DPBS supplemented with calcium and magnesium (14040133; Gibco, Grand Island, USA). LN511 and LN521 are human recombinant laminins that are produced as animal-origin free components. Then, the ECM protein solution was added to tissue culture polystyrene (TCPS) dishes and incubated overnight at 4°C. The excess ECM protein solution in the TCPS dishes was discarded, and the ECM protein-coated TCPS dishes were used for the following experiments.

### Cell lines and hPSC culture

hiPSCs (HPS0077, female) were purchased from Riken BioResource Center (Tsukuba, Japan). hiPSCs (Mix-2) were prepared from reprogrammed human amniotic stem cells used in our previous study [61]. hESCs (H9, female) were obtained from WiCell Research Institute (Madison, WI, USA). These hPSCs were selected as representative hPSCs for differentiation into RPE cells in this study. The cells were cultivated on Matrigel-coated dishes or ECM protein-coated dishes in mTeSR1 medium (85850, Stemcell, Vancouver, Canada) at 37°C (5% CO_2_ humidified air). The cell culture medium was replaced every day, and the cells were passaged every 4–5 days using Dispase II (D4693, Sigma, Darmstadt, Germany). The other chemicals, proteins and biomaterials used in the present work are listed in Supplementary Table S1.

### hPSC cell counting

Cell counting was performed to ensure a uniform seeding density in each experiment. First, a cell suspension was prepared by using Accutase solution. Then, a small amount of cells (10 μl) was aspirated and inserted into a hemocytometer. The cells were visualized using an automatic cell counter (DeNovix, CellDrop BF, Wilmington, USA).

### Generation and passage of RPE cells from hPSCs using the activin A protocol

hPSCs were cultured on LN511-, LN521-, rVN- and Matrigel-coated dishes and were treated with N2B27 medium for 1 week until the confluence of the cells reached 80–90%. The N2B27 medium consisted of 50% DMEM/F12, GultaMAX, 50% neurobasal medium, 0.5× B27 supplement, 0.5× N2 supplement, 55 μM 2-mercaptoethanol and 1× L-glutamine (Figure 1B). For the weekly medium change, 1.5 ml of culture medium per 3.5 cm^2^ dish was changed on Day 0 and Day 2, and then 2 ml of culture medium was changed on Day 4. Subsequently, the cells were washed twice in DPBS without calcium or magnesium and cultured in 1.5 ml of RPE medium supplemented with 50 ng/ml recombinant human activin A on Day 7 (Figure 1B). RPE medium was prepared as follows: DMEM (high glucose), 10% KnockOut Serum Replacement (KOSR), 1× L-glutamine, 1× MEM nonessential amino acid (MEM NEAA) solution, 55 mM 2-mercaptoethanol and 1× penicillin–streptomycin. RPE medium supplemented with 50 ng/ml recombinant human activin A was changed weekly until the first pigmentation was observed by the naked eye (approximately 18–21 days). Once pigmentation could be observed, activin A was removed from the culture medium. Only RPE medium was used for further induction of RPE (Figure 1B). When 35–40% of the culture plate surface showed pigmentation at approximately Day 35, the cells were passaged for expansion.

The RPE cells obtained by this protocol were tightly connected to each other. Therefore, the cells were passaged using the following methods. The cells were washed in DPBS and resuspended in Accutase solution for 10–20 min at 37°C after being scraped off the dish with a cell scraper. The cells were centrifuged at 200× *g* for 5 min to remove the supernatant, after which a single-cell suspension was obtained. The cells were then resuspended in RPE medium containing 10% KOSR. Cells were seeded at a rate of 500 000 cells/cm^2^ in dishes coated with fresh ECM proteins. After the cells were successfully passaged, the RPE medium supplemented with 10% KOSR was changed every day to maintain cell growth from Day 35 to Day 38. Furthermore, RPE medium supplemented with 5% KOSR was added following the weekly routine of changing the medium for further cultivation.

### Generation and passage of RPE cells from hPSCs using the NIC84 protocol

The NIC84 protocol developed by Smith *et al.* [31] was modified for use in this study (Figure 1C). hPSCs (20 000 cells/cm^2^) were seeded individually on LN511-, LN521-, rVN- and Matrigel-coated dishes and cultivated in mTesR1 medium until the hPSCs reached 100% confluence (approximately 5 days). Subsequently, 2 ml of prewarmed differentiated medium (DM) was added to 3.5 cm^2^ ECM protein-coated TCPS dishes on Day 0 for RPE differentiation. The DM mixture consisted of 85% DMEM/F12, 15% KOSR, 2 mM GlutaMAX supplement, 0.1 mM MEM-NEAA and 1% Antibiotic-Antimycotic. Then, the medium was replaced with 2 ml of prewarmed DM supplemented with 10 mM NIC and 10 nM CTM on Day 2, and the medium was changed to 2 ml of prewarmed DM supplemented with 10 mM NIC and 20 nM CTM on Day 3 (Figure 1C). Subsequently, the medium was replaced with 2 ml of prewarmed DM supplemented with 10 mM NIC and 30 nM CTM every day from Day 4 to Day 14 (Figure 1C). Starting on Day 14, only DM supplemented with 10 mM NIC was added to the cell culture medium to maintain RPE induction until Day 28, after which the medium was replaced every day (Figure 1C). On Day 28, the cells were passaged for cell expansion. The cell passage process was as follows.

The cells were washed twice with DPBS. One milliliter of Accutase solution was added to a 3.5-cm^2^ dish in a 37°C incubator for 10–15 min until the cells were rounded. The cells were dissociated in Accutase solution with prewarmed DM until the clumps disappeared. The cells were centrifuged at 200 ×*g* for 5 min, and the cells were resuspended in medium composed of prewarmed 50% DM and 50% RPEMN to support cell attachment to the dishes. The cells were seeded at a density of 250 000–300 000 cells/cm^2^ in ECM protein-coated TCPS dishes. After passage, the cells were incubated in RPEMN medium for 29 days. The RPEMN medium consisted of 70% DMEM, 30% F12, 2% B27 supplement and 1% L-glutamine. The RPEMN medium was subsequently changed every day from Day 30 to Day 56. After 56 days, the cells were passaged again. Subsequently, the cells were cultured in RPEMN medium to maintain RPE induction from Day 57 to Day 84 (Figure 1C). The medium was changed every day.

### Immunofluorescence staining of hPSC-derived RPE cells

The cells were passaged on glass bottom culture dishes for laser confocal scanning microscopy (LSM900 with Airyscan, Zeiss, Germany). The cells were washed with PBS, fixed in 4% paraformaldehyde for 15 min at room temperature (RT), permeabilized with 0.1% Triton X-100 for 15 min at RT, and blocked in blocking buffer for 30 min at RT. Then, the cells were incubated with primary antibodies (antibodies against PAX6, ZO-1, MITF and RPE65) overnight at 4°C in Antibody Dilution Buffer (P0103; Beyotime, Shanghai, China). Subsequently, the cells were washed three times with DPBS the next day and then incubated with secondary antibodies diluted in antibody dilution buffer for 1 h at 37°C in the dark. The cells were washed again with DPBS. Nuclei were stained with DAPI. The antibodies used in this study are listed in Supplementary Table S2.

### Flow cytometry assay of hPSC-derived RPE cells

The cells were detached from the dishes and washed twice with washing buffer. Then, the cells were fixed with 1% paraformaldehyde and incubated at RT for 20 min. Subsequently, the cells were treated with 90% cold methanol and incubated at 4°C for 15 min. After centrifugation (200× g), the cells were resuspended in DPBS and divided into different tubes. Then, the isotype control antibody or primary antibody (antibodies against PAX6, ZO-1, MITF or RPE65) was added to each tube, all of which were incubated at RT for 1 h. The cells were subsequently washed with washing buffer, and secondary antibodies were added to the washed cells. Subsequently, the cells were incubated at RT for 30 min in the dark. Then, washing buffer was added to the tubes, and the tubes were centrifuged at 200× g for 5 min. The washing step was repeated three times. Finally, the cells stained with specific antibodies were analyzed using flow cytometry (BD AccuriTM C6, BD Biosciences, Franklin Lakes, NJ, USA). The antibodies used in the flow cytometry assay are listed in Supplementary Table S2.

### Subretinal transplantation of cells into RCS rats

Royal College of Surgeons (RCS) rats (an animal model for retinal degeneration) were used in this study and all animal experiments complied with the ARRIVE guidelines. The animal experiments were approved by the Laboratory Animal Ethics Committee of Wenzhou Medical University (No. wydw2021-0230) and all procedures were designed based on the guidelines of the Laboratory Animal Center of Wenzhou Medical University. hPSC-derived RPE cells (5 × 10^4^/μl) were labeled with CellTracker Green CMFDA Dye. At postnatal Day 21, both male and female littermates of RCS rats were assigned randomly to different transplantation groups. These groups included subretinal transplantation of HPS0077-derived RPE cells (differentiated on Matrigel-coated dishes), HPS0077-derived RPE cells (differentiated on LN521-coated dishes) and a blank control group (subretinal injection of PBS), a total of 10, 9 and 6 rats were used for OMR analysis, respectively, and 9, 7 and 5 rats were used for ERG analysis 4 weeks after cell transplantation, respectively, and 8, 6 and 5 rats were used for ERG analysis 8 weeks after cell transplantation, respectively. 1% sodium pentobarbital (30 mg/kg) was injected intraperitoneally to anesthetize the rats. Tropicamide eye drops were used to induce mydriasis in RCS rats, proparacaine hydrochloride eye drops were used for ocular surface anesthesia, and ofloxacin eye ointment was used for ocular surface protection [62].

Subsequently, the RCS rats were subjected to cell transplantation under an operating microscope (M620F20; Leica Microsystems, Wetzlar, Germany). A 29G needle (BD, cat. no. 328421) was used to establish a path 1 mm outside the limbus. Two microliters of the cell suspension were then uniformly transplanted into the subretinal space of the right eye using a 33G Hamilton blunt needle and syringe. After cell transplantation, 0.2 g/l cyclosporine A (an immunosuppressive medicine) was added to the drinking water of the rats in each group. Immediately after cell transplantation into RCS rats, photographs of the ocular fundi of RCS rats were taken using an IISCIENCE (EYEMERA, Yangsan, Korea) retinal imaging microscope to determine whether the cell transplantation was successful. Subretinal filtering blebs were formed, and green, fluorescent blebs (cell suspension injection group) appeared after successful injection [62].

### Optical coherence tomography

Optical coherence tomography (OCT) is a noninvasive diagnostic technique that provides an *in vivo* cross-sectional view of the retina [63]. Before OCT imaging, the RCS rats were anesthetized with sodium pentobarbital, and mydriasis was induced by tropicamide eye drops. The corneal surface was protected using hydroxyethylcellulose solution. OCT was performed using a Spectralis OCT (Heidelberg Engineering, Franklin, USA). The ocular fundus of each rat was monitored using a fundus camera. Representative OCT images of the retina were taken horizontally across the optic nerve head (ONH), and the imaging location is marked on the image with a green line.

### Optomotor response test

We evaluated the optomotor response (OMR) with the use of a commercial qOMR system (PhenoSys, Berlin, Germany) that utilizes automated head tracking and behavioral analysis [64]. RCS rats were placed on a platform and stimulated with a rotating (12·s^−1^) vertical sinusoidal grating at different spatial frequencies. The contrast between black and white was set to 100%. The spatial frequencies included 0.05, 0.1, 0.15, 0.20, 0.25, 0.30, 0.35, 0.375, 0.40, 0.425, 0.45 and 0.5 cycles/degree, which were altered in a random order every minute. Based on real-time head tracking, quantitative OMR measurements automatically provided visual acuity and contrast sensitivity.

### Electroretinograms

Electroretinograms (ERGs) were recorded using an electrophysiology system (RETI-Port21, Roland, Germany). After at least 12 h of dark adaptation, the RCS rats were anesthetized, and pupil dilation was performed using tropicamide phenylephrine. The RCS rats were placed on a stationary heating pad throughout the experiment. ERG signals were recorded through a gold ring with eye ointment placed in contact with the corneal surface. In addition, needle electrodes were inserted into the cheeks and tails of the RCS rats to serve as reference and ground leads, respectively. The dark test was performed with eight stimulus response sequences (dark 0.01, 3.0 and 10.0 cd/m^2^ and light 3.0 in sequence). The resulting *b*-wave amplitude was measured as reported previously from the a-wave trough to the b-wave peak [65].

### Statistical analysis

The experimental data are presented as the mean ± standard deviation (SD). The quantitative data of the samples were analyzed by ANOVA. OMR results were performed using the Tukey test for multiple comparisons after ANOVA, and ERG results were performed using the Fisher’s LSD test for multiple comparisons after ANOVA. Excel (Microsoft, Redmond, WA, USA) and GraphPad Prism 9.0 software (GraphPad Software, Inc., La Jolla, CA) were used for data analysis and mapping, and *P *<* *0.05 was considered to indicate statistical significance.

## Results

### hiPSC differentiation into RPE cells on xeno-free ECM protein-coated dishes using the activin A protocol

**Figure 2.**
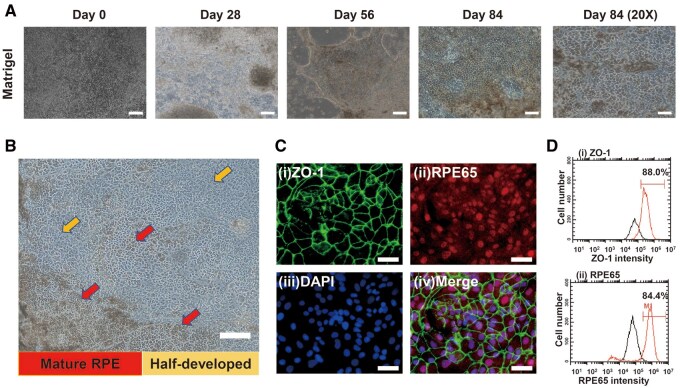
Generation of hiPSC-derived RPE cells using the activin a protocol. (**A**) The morphology of hiPSC-RPE cells cultured on Matrigel-coated dishes on Day 0, Day 28, Day 56, and Day 84. Scale bar: 100 μm. (**B**) hiPSC**-**RPE cells at different maturity levels were seeded on Matrigel-coated dishes. Mature RPE cells are indicated by red arrows, and immature RPE cells are indicated by yellow arrows. Scale bar: 200 μm. (**C**) Expression of RPE markers (ZO-1 (i) and RPE65 (ii)) in hiPSC-RPE cells cultured on Matrigel-coated dishes determined by immunostaining on Day 84. Nuclei were stained with DAPI (iii). The photo in (iv) was created by merging (i) to (iii). Scale bar: 100 μm. (**D**) Expression of RPE markers (ZO-1 (i) and RPE65 (ii)) in hiPSC-RPE cells cultured on Matrigel-coated dishes was assessed via flow cytometry on Day 84. The peak with relatively low intensity (black line) represents the cells stained with the isotype antibody.

We differentiated the hiPSCs (HPS0077) into RPE cells in Matrigel-coated dishes according to the original activin A protocol (Figure 1B). Eventually, the cells outgrew their original cystic structure and began to produce pigmented cells at the edges around Day 56 (Figure 2A). Many homogeneous pebble stone cells appeared in the dishes after 84 days of induced differentiation (Figure 2A). However, there were still other cells at various degrees of differentiation; we divided them into mature RPE cells (indicated by red arrows) and immature RPE cells (indicated by yellow arrows) for further purification (Figure 2B). The protein expression of the hiPSC-derived RPE cells (after 84 days of differentiation) was assessed via an immunofluorescence assay (Figure 2C) and flow cytometry (Figure 2D). The mature RPE cell marker RPE65 (84%) and the structural marker ZO-1 (88%) were strongly expressed as detected by flow cytometry (Figure 2D).

Subsequently, we evaluated the effect of ECM protein-coated dishes on hiPSC differentiation into RPE cells following the original activin A protocol (Supplementary Figure S1). On Day 28, the RPE cell marker expression of the remaining cells on Matrigel-, rVN-, LN511- and LN521-coated dishes was evaluated by flow cytometry (Supplementary Figure S2). Surprisingly, the RPE-related markers PAX6 (96%), MITF (88%), ZO1 (41%) and RPE65 (67%) were highly expressed on hiPSC-derived RPE cells differentiated on Matrigel-coated dishes even after 28 days of differentiation. Unfortunately, all the cells that differentiated on the ECM protein-coated dishes eventually detached at approximately Day 30 after passage (Supplementary Figure S1A).

Then, we adjusted the concentration of activin A from 50 to 25 ng/ml, on Day 13, polygonal cells were observed on the Matrigel-, LN521- and LN511-coated dishes (Supplementary Figure S1B). Encouragingly, the pigmented cells were observed on Matrigel-coated dishes on Day 19 and on LN511-coated dishes on Day 22 (Supplementary Figure S1C). However, all the cells cultured on any of the ECM-coated dishes except for the Matrigel-coated dishes still died after passage. Therefore, we subsequently investigated the use of another differentiation protocol, the NIC84 protocol, for hiPSC differentiation on our several types of ECM protein-coated dishes.

### Preparation of hiPSC-derived RPE cells using a modified NIC84 protocol

The NIC84 protocol published by Maruotti *et al.* [30] and verified by Smith *et al.* [31] was modified for use in this study (Figure 1C). When 30 nM CTM was added to the medium in this study, the cells were able to attach to the ECM protein-coated dishes successfully through Day 28. However, after the cells were passaged on Day 28, many cells detached and eventually died within a week. Therefore, we gradually increased the concentration of CTM (by 10 nM every 3-day interval) to 30 nM, to prevent drastic environmental changes to the cells. Subsequently, the medium was changed to mixed medium containing DM (50%) with RPEMN (50%) on Day 29 when the cells became more stable.

**Figure 3.**
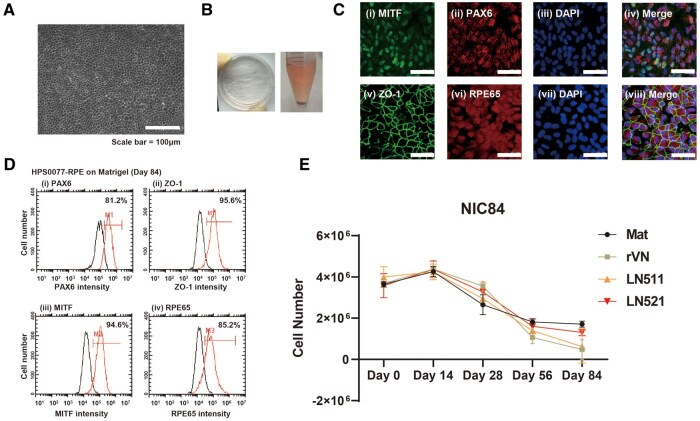
Generation of hiPSC-derived RPE cells using the modified NIC84 protocol. (**A**) Characteristic polygonal structure of hiPSC-RPE cells cultured on Matrigel-coated dishes. Scale bar: 100 μm. (**B**) The black precipitate of hiPSC-RPE cells cultured on Matrigel-coated dishes. (**C**) Expression of the RPE cell markers MITF (i), PAX6 (ii), ZO-1 (v) and RPE65 (vi) in hiPSC-RPE cells cultured on Matrigel-coated dishes was assessed by immunostaining on Day 84. Nuclei were stained with DAPI (iii, vii). the photos in (iv) and (viii) were created by merging (i) to (iii) and (v) to (vii), respectively. Scale bar: 100 μm. (**D**) Expression of the RPE cell markers PAX6 (i), ZO-1 (ii), MITF (iii) and RPE65 (iv) in hiPSC-RPE cells cultured on Matrigel-coated dishes determined via flow cytometry on Day 84. The peak with relatively low intensity (black line) represents the cells stained with the isotype antibody. (**E**) The number of hiPSC-RPE cells (per ml) during differentiation on Matrigel-, rVN-, LN511- and LN521-coated dishes on days 0, 14, 28, 56 and 84.

The modified NIC84 protocol (Figure 1C) was used to successfully induce the differentiation of hiPSCs into mature RPE cells with characteristic pebble-like morphologies on Matrigel-coated dishes (Figure 3A). At the end of the induction period (images were taken at 84 days of differentiation), many pigmented cells appeared (Figure 3B). In addition, on Day 84, the hiPSC-derived RPE cells induced on the Matrigel-coated dishes strongly expressed the RPE-related markers PAX6, ZO1, MITF and RPE65, as detected by immunostaining (Figure 3C). Flow cytometry analysis of the expression of the RPE cell markers PAX6 (81%), MITF (94%), ZO1 (95%) and RPE65 (85%) by flow cytometry revealed consistent results with those from immunofluorescence (Figure 3C and D).

### Morphology and cellular proliferation rates of hiPSC-derived RPE cells cultured on different ECM protein-coated dishes using the modified NIC84 protocol

**Figure 4.**
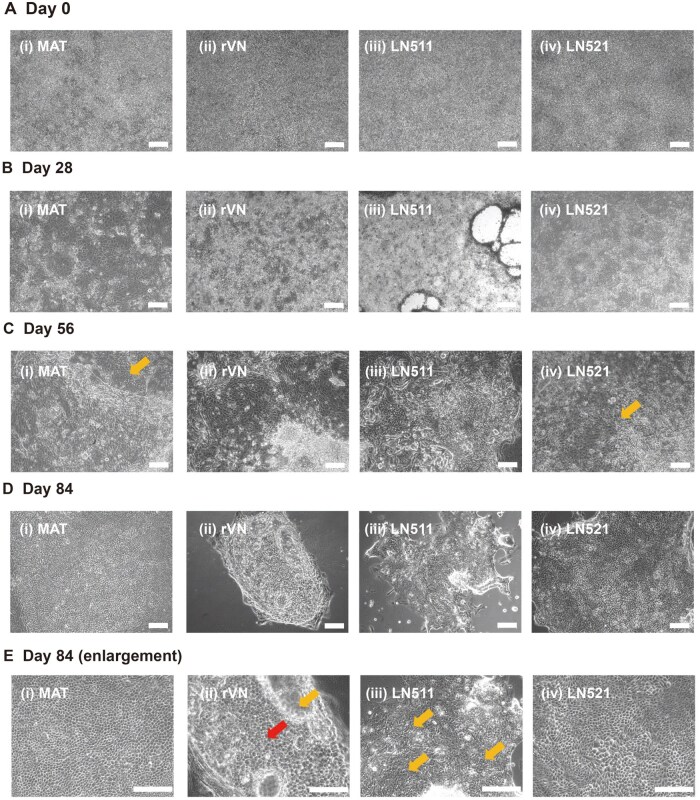
The morphology of hiPSC-RPE cells cultured on Matrigel (i), rVN (ii), LN511 (iii) and LN521 (iv)-coated dishes on Day 0 (**A**), Day 28 (**B**), Day 56 (**C**) and Day 84 (**D**) using the modified NIC84 protocol. (**E**) The enlarged view of (**D**) shows the cobblestone structure of the mature RPE. Mature RPE cells (central red arrow of E(ii)) have a polygonal stereostructure, and immature RPE cells (remaining yellow arrows) mostly present a flat structure. Scale bar: 100 μm.

We investigated whether mature RPE cells could be successfully induced from hiPSCs (HPS0077) by following the modified NIC84 protocol (Figure 1C) in Matrigel-coated dishes or xeno-free ECM-coated dishes (Figures 3E and 4A–E).

The cells cultured on Matrigel- and LN521-coated culture dishes exhibited the most polygonal structures on Day 28 (Figure 4B(i) and (iv)). However, cells cultured on rVN-coated dishes often needed to be passaged on Day 21 to replace the rVN-coated dishes and to prevent cell detachment. Although LN511 has been reported to have good adhesion to neural cells [66], cell shedding was observed in this study on LN511-coated dishes on Day 28 (Figure 4B(iii)). The cells could be expanded after being passaged on the freshly LN511-coated dish on Day 28. The cells on the rVN- and LN511-coated dishes also exhibited many polygonal structures on Day 56, although there were still some non-RPE cells on the dishes (Figure 4C(ii) and (iii)). Notably, the RPE-like cells exhibited more stereoscopic morphologies on the Matrigel- and LN521-coated dishes than on the other dishes, whereas some mature RPE cells and cells with dome structures formed on those dishes (Figure 4C(i) and (iv)). These observations provide favorable evidence that hiPSCs (HPS0077) successfully become mature RPE cells on some ECM-related protein-coated dishes.

Many cells with homogeneous polygon structures and typically brown or black pigment structures in the RPE appeared on the Matrigel- and LN521-coated dishes on Day 84 (Figure 4D(i) and (iv)), indicating the production of pigmented cells with increased purity (Figure 4E(i) and (iv)). Cells with polygonal features appeared after 4 weeks of differentiation, and most cells turned black by Day 84. The deposition of melanin on the surface of the Matrigel- and LN521-coated dishes was visible to the naked eye on Day 84 (Supplementary Figure S3A and B). In addition, progressive changes in the color or morphology of the cells were observed from Day 14 to Day 84; these changes are important indicators of the successful differentiation of hiPSCs into RPE cells (Supplementary Figure S3C).

The proliferation of hiPSC-derived RPE cells was evaluated to determine the most suitable type of ECM protein-coated dish for RPE differentiation and cultivation (Figure 3E). The seeding densities of cells cultured on several ECM protein-coated dishes were the same at the beginning (20 000 cells/cm^2^), and the cells were cultured until they reached confluence (Figure 4A). The cell proliferation rates exhibited a decreasing trend after Day 14 on each of the ECM protein-coated dishes (Figure 3E). Subsequently, the cell proliferation rate decreased slightly from Day 28 to Day 84 on the Matrigel- and LN521-coated dishes, which indicated that the LN521-coated dishes provided stable substrates for RPE differentiation and cultivation, similar to the Matrigel-coated dishes. On the other hand, complete cell detachment occurred on most of the LN511- and rVN-coated TCPS dishes owing to the failure of the hiPSCs to differentiate into mature RPE cells from Day 28 to Day 84 of differentiation (Figure 3E). Therefore, LN521-coated dishes, which are considered xeno-free cell culture dishes, were the most suitable for the differentiation of hiPSCs into RPE cells.

### Differentiation of other hPSC lines into RPE cells using a modified NIC84 protocol

We evaluated the ability of other hPSC lines to differentiate into RPE cells using our modified version of the NIC84 protocol. H9 hESCs (Supplementary Figure S4A) and our custom-made hiPSCs, Mix-2 (Supplementary Figure S4B), were selected for evaluation of the hPSC differentiation into RPE cells on Matrigel-coated dishes. The marker expression of H9-derived RPE cells (Supplementary Figure S4C) or Mix2-derived RPE cells (Supplementary Figure S4D) (after 84 days of differentiation) was evaluated via an immunofluorescence assay. The mature RPE marker RPE65 in H9-derived RPE cells (55%) and MIX2-derived RPE cells (90%) was identified by flow cytometry after 84 days of differentiation (Supplementary Figure S4E and F).

### Immunostaining of hiPSC-derived RPE cells on several types of ECM protein-coated dishes

**Figure 5.**
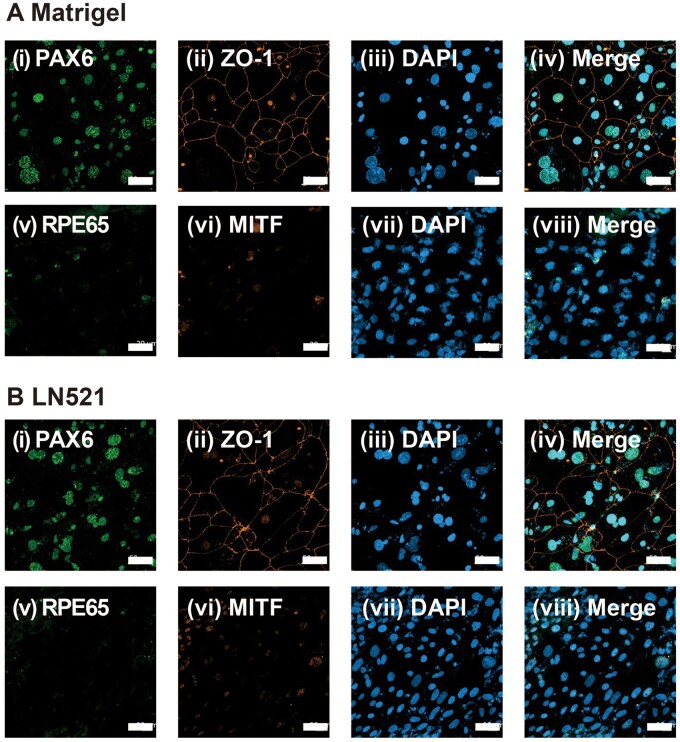
Expression of the RPE cell markers PAX6 (i), ZO-1 (ii), RPE65 (v) and MITF (vi) in hiPSC-derived RPE cells cultured on Matrigel (**A**)- and LN521 (**B**)-coated dishes by immunostaining on Day 28 following the modified NIC84 protocol. Nuclei were stained with DAPI (iii, vii). The photos in (iv) and (viii) were created by merging (i) to (iii) and (v) to (vii), respectively. Scale bar: 50 μm.

**Figure 6.**
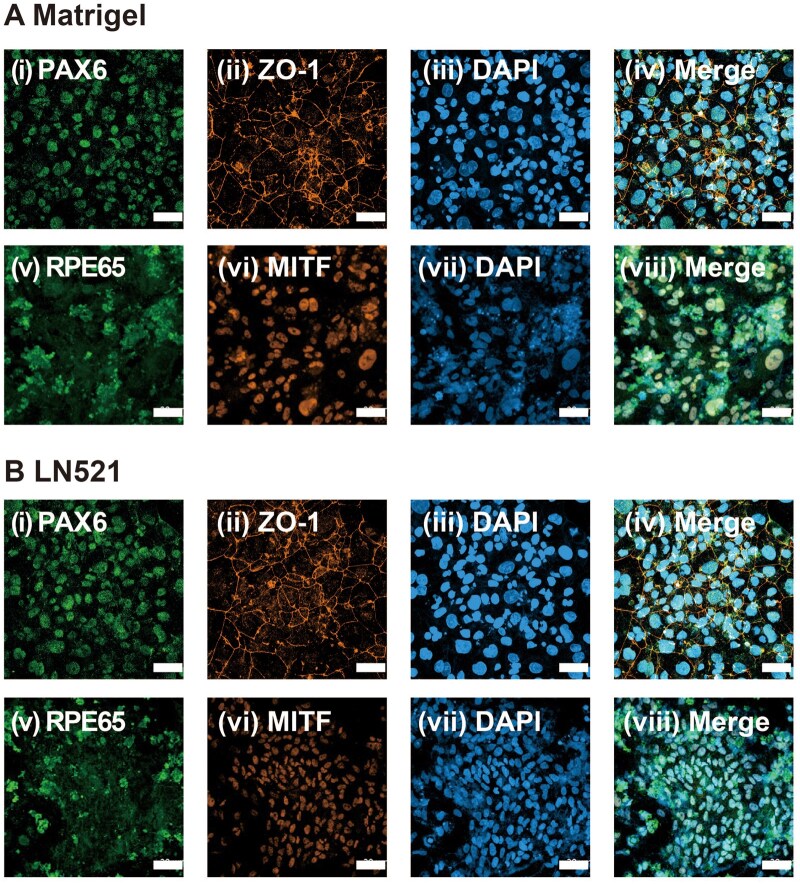
Expression of the RPE cell markers PAX6 (i), ZO-1 (ii), RPE65 (v) and MITF (vi) in hiPSC-derived RPE cells cultured on Matrigel (**A**)- and LN521 (**B**)-coated dishes by immunostaining on Day 56 following the modified NIC84 protocol. Nuclei were stained with DAPI (iii, vii). The photos in (iv) and (viii) were created by merging (i) to (iii) and (v) to (vii), respectively. Scale bar: 50 μm.

**Figure 7.**
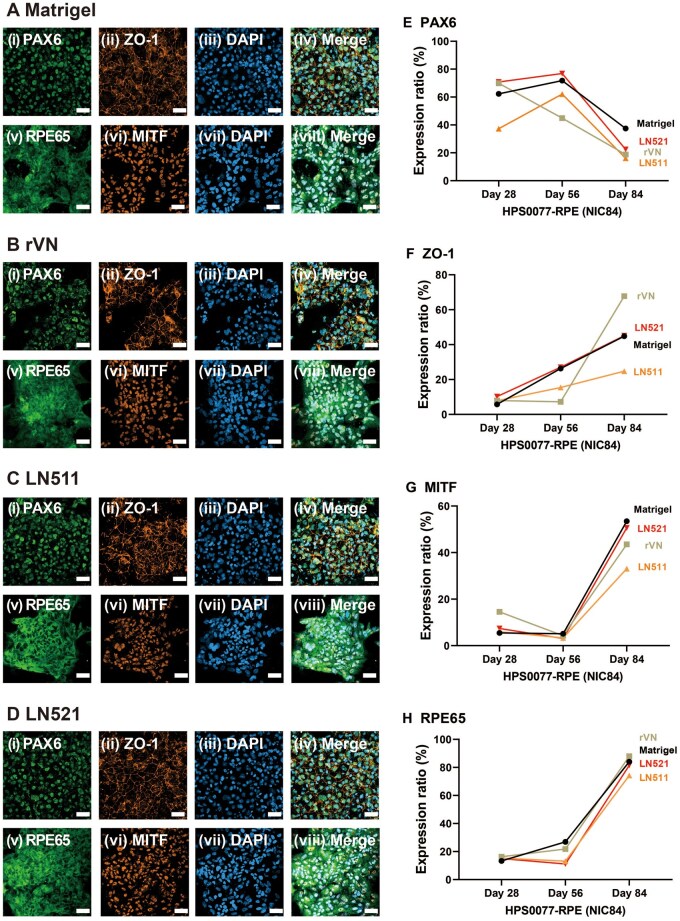
Expression of the RPE cell markers PAX6 (i), ZO-1 (ii), RPE65 (v) and MITF (vi) in hiPSC-derived RPE cells cultured on Matrigel- (**A**), rVN- (**B**), LN511- (**C**) and LN521- (**D**)-coated dishes on Day 84 following the modified NIC84 protocol. Nuclei were stained with DAPI (iii, vii). The photos in (iv) and (viii) were created by merging (i) to (iii) and (v) to (vii), respectively. Scale bar: 50 μm. The expression of the RPE markers PAX6 (**E**), ZO-1 (**F**), MITF (**G**) and RPE65 (**H**) in Matrigel-, rVN-, LN511- and LN521-coated dishes was determined via flow cytometry on days 28, 56 and 84 following the modified NIC84 protocol.

The expression of RPE cell markers on hiPSC (HPS0077)-derived RPE cells was evaluated at different time points using an immunostaining method to monitor the growth trajectory of RPE cells induced on several ECM protein-coated dishes via our modified NIC84 protocol, and the results are shown in Figures 5–7 and Supplementary Figures S5 and S6. PAX6 and ZO1 were expressed on Day 28 in each of the ECM protein-coated dishes (Figure 5A(i) and (ii) and Supplementary Figure S5A(i) and (ii) and B(i) and (ii)), while MITF and RPE65 were scarcely expressed (Figure 5A(v) and (vi) and Supplementary Figure S5A(v) and (vi) and B(v) and (vi)). Surprisingly, on Day 28, the cells expressed the RPE structural marker ZO-1, which indicated that the cells formed tight junctions and that hiPSC-derived RPE cells were efficiently generated by using our modified NIC84 protocol. The expression of the RPE-associated markers RPE65 and MITF was also detected on Day 56 of differentiation. The results showed that the cells strongly expressed PAX6 and ZO1 (Figure 6A(i) and (ii) and Supplementary Figure S6A(i) and (ii) and B(i) and (ii)), whereas the cells weakly expressed RPE65 and MITF (Figure 6A(v) and (vi) and Supplementary Figure S6A(v) and (vi) and B(v) and (vi)). According to the immunofluorescence assay, the expression of RPE65 and MITF increased significantly after Day 84 of differentiation compared to Day 56 in the cells cultured on Matrigel-, rVN-, LN511- and LN521-coated dishes, which indicated that mature hiPSC-derived RPE cells were generated (Figure 7A(v), (vi), B(v), (vi), C(v), (vi), D(v) and (vi)). Remarkably, stronger RPE65 expression was observed in the cytoplasm of cells cultured on Matrigel- and LN521-coated dishes than in the cytoplasm of cells cultured on rVN- and LN511-coated dishes on Day 84 (Figure 7A(v) and D(v)).

### Quantitative analysis of hiPSC-derived RPE cell markers in several types of ECM protein-coated dishes by flow cytometry

The expression of RPE cell markers on hiPSC (HPS0077)-derived RPE cells in several ECM protein-coated dishes was analyzed by flow cytometry to quantitatively detect the expression of specific RPE cell markers. PAX6 was expressed in 62%, 69%, 37% and 70% of the cells cultured on Matrigel-, rVN-, LN511- and LN521-coated dishes, respectively, as determined after 28 days of cell differentiation by flow cytometry (Figure 7E and Supplementary Figure S7A(i), B(i), C(i) and D(i)). However, very low expression of ZO1, MITF and RPE65 was detected in the cells at that time (Figure 7F–H and Supplementary Figure S7A(ii)–(iv), B(ii)–(iv), C(ii)–(iv) and D(ii)–(iv)).

According to flow cytometry, PAX6 was expressed in 71%, 44%, 62%, and 76% of the differentiated cells after 56 days of differentiation; these cells were cultured on Matrigel-, rVN-, LN511- and LN521-coated dishes, respectively (Figure 7E and Supplementary Figure S8A(i), B(i), C(i) and D(i)). Furthermore, flow cytometry revealed that 26%, 7.3%, 15%, and 27% of the differentiated cells expressed ZO1 after they were cultured on Matrigel-, rVN-, LN511- and LN521-coated dishes, respectively, for 56 days of differentiation (Figures 7G and Supplementary Figure S8A(ii), B(ii), C(ii) and D(ii)). The expression rates were much higher on Day 56 than on Day 28, and the expression tendency was consistent with the tendency observed using immunofluorescence (Figures 5 and 6 and Supplementary Figures S5 and S6).

The expression of RPE65 began to increase on Day 56 of differentiation (Figure 7H and Supplementary Figure S8A(iv), B(iv), C(iv) and D(iv)), whereas MITF expression was still scarcely detected (Figure 7F and Supplementary Figure S8A(iii), B(iii), C(iii) and D(iii)). Notably, 26% of the cells on Matrigel-coated dishes expressed the mature RPE marker RPE65 on Day 56 of differentiation according to the flow cytometry assay (Figure 7H). The percentage of RPE65-expressing cells cultured on rVN-coated dishes was 21% on Day 56 of differentiation, which was slightly lower than that on Matrigel-coated dishes (Figure 7H).hiPSC-derived RPE cells differentiated on ECM protein-coated dishes showed high expression levels of RPE65 (84%, 88%, 74% and 81%), which were differentiated on Matrigel-, rVN-, LN511- and LN521-coated dishes, respectively, on Day 84 of differentiation (Figure 7H and Supplementary Figure S9A(iv), B(iv), C(iv) and D(iv)). These findings were analyzed by flow cytometry, which indicated that the modified NIC84 protocol, which was developed during this study, could generate mature RPE cells on several ECM protein-coated dishes after approximately 84 days (3 months). Notably, the expression levels of each of the four RPE cell markers, PAX6, ZO1, MITF and RPE65, were 16%, 24%, 33% and 74%, respectively, on Day 84 of differentiation in the differentiated cells that were cultured on LN511-coated dishes; these were the lowest rates of expression among the cells cultured on ECM protein-coated dishes investigated in this study (Figure 7E–H and Supplementary Figure S9C(i)–(iv)).

In summary, the expression of ZO1, MITF and RPE65 on differentiated cells increased with increasing differentiation time (Figure 7F–H), whereas the expression of PAX6, an early marker of the eye field, increased linearly with increasing differentiation time (Figure 7E). These results suggest that mature hiPSC-derived RPE cells could be generated after approximately 84 days of differentiation not only on Matrigel-coated dishes but also on some ECM protein-coated dishes, such as LN521-coated dishes, using the modified NIC84 protocol.

### Visual recovery by transplantation of hiPSC-derived RPE cells into RCS rats

**Figure 8.**
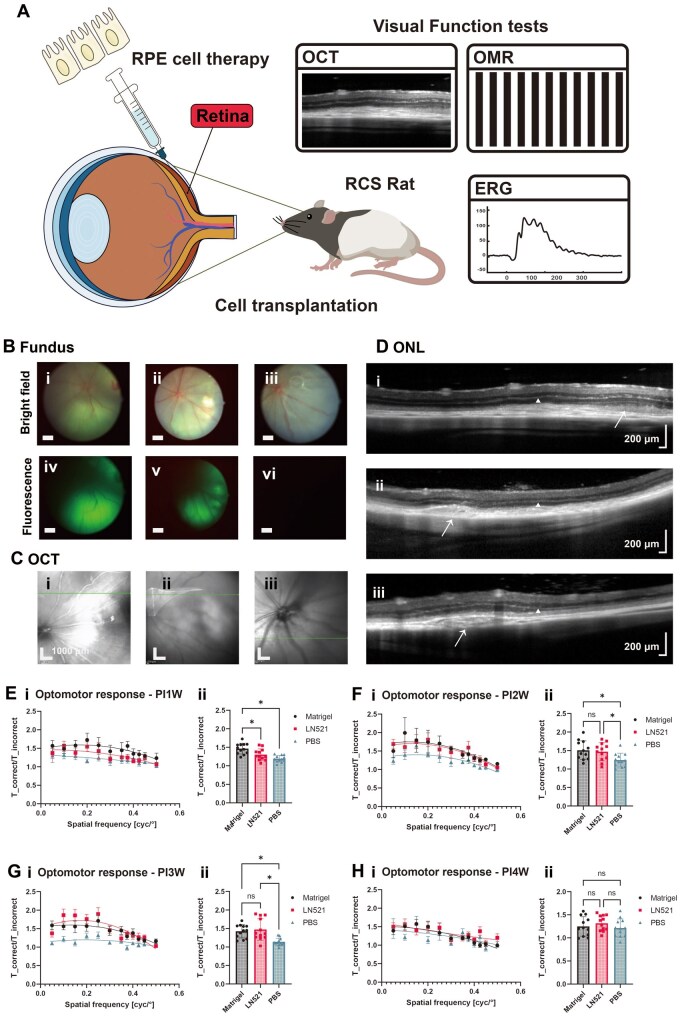
Subretinal transplantation of hiPSC-RPE cells and visual functional examination of RCS rats. (**A**) An illustration of the subretinal transplantation of hiPSC-RPE cells and visual functional examination of RCS rats. (**B**) Fundus imaging in RCS rats immediately after cell transplantation (differentiated on Matrigel-coated (i, iv) and LN521-coated (ii, v) dishes) or PBS buffer (iii, vi) injection. (**C** and **D**) OCT images at 4 weeks after cell transplantation or PBS injection. The images represent the retinal condition after the transplantation of cells differentiated on Matrigel-coated (C (i), D (i)) or LN521-coated (C (ii), D (ii)) dishes or injected with PBS buffer (C (iii), D (iii)). The white arrow indicates the location of the transplant, and the arrowhead indicates the ONL. (**E**–**H**) Visual acuity of RCS rats at 1 week (E), 2 weeks (F), 3 weeks (G) and 4 weeks (H) after cell transplantation (differentiated on Matrigel-coated (*n* = 10) and LN521-coated (*n* = 9) dishes) or PBS injection (*n* = 6)), as measured by qOMR. The data are presented as the means ± SDs. Statistical analysis of the visual acuity of RCS rats after cell transplantation at 1 week (E (ii)), 2 weeks (F (ii)), 3 weeks (G (ii)) and 4 weeks (H (ii))) was performed after fitting using second-order quadratic polynomials (1 week E (i)), 2 weeks (F (i)), 3 weeks (G (i)) and 4 weeks (H (i)). *P <* *0.05.

hiPSC (HPS0077)-derived RPE cell (labeled with green fluorescence using Cell Tracker) suspensions, which were differentiated on Matrigel-coated and LN521-coated dishes, were subretinally transplanted into the right eyes of 21-day-old RCS rats (Figure 8A). Fundus photography was used to observe the ocular fundus of RCS rats immediately after cell transplantation, and the results are shown in Figure 8B. The results showed that the fundi were normal without bleeding or obvious leakage of cells or solution, which was indicated by a transparent space in the injection area (Figure 8B (i)–(iii)). On the fundus photograph, the RCS rats subjected to PBS showed retinal eminence without green fluorescence (Figure 8B(vi)), whereas the RCS rats subjected to cell suspension injection exhibited obvious green fluorescence (Figure 8B(iv) and (v)), which suggested that the hiPSC-derived RPE cell suspension and PBS were successfully injected into the subretinal space of the RCS rats.

We used OCT [67] to analyze the eyes of RCS rats to evaluate whether the transplantation of hiPSC-derived RPE cells was effective at improving the outer nuclear layer (ONL) thickness, and the results are shown in Figure 8C and D. The positions of the SD-OCT scans in the image are shown in Figure 8D(i)–(iii), and the corresponding green lines are marked in the image shown in Figure 8C(i)–(iii). The transplantation position (white arrow) could still be observed on the OCT images 4 weeks after injection (Figure 8D(i)–(iii)); the ONL (asterisks in Figure 8D(i)–(iii)) was more pronounced in the cell transplantation group than in the PBS injection group (negative control) (Figure 8D(i)–(iii)). The average ONL thickness was greater in the rats in the cell transplantation group (Figure 8D(i) and (ii)), for which the substrates were differentiated on Matrigel-coated and LN521-coated dishes, respectively, than in the PBS injection group (Figure 8D(iii)). Interestingly, the ONL maintained a large thickness near the PBS injection site even in the PBS injection group. This could be caused by the physical effect of the needle injection.

We used optomotor response/reflex (OMR) assays [68] to detect the optomotor response of RCS rats at 1, 2, 3 and 4 weeks after transplantation of hiPSC-derived RPE cells (Figure 8E and F). There were significant differences 2 and 3 weeks after transplantation in the optomotor responses of RCS rats subjected to transplantation of hiPSC-derived RPE cells that were differentiated on Matrigel- and LN521-coated dishes compared to those of RCS rats that received PBS injection (Figure 8F(ii) and G(ii)). In addition, the qOMRs of RCS rats transplanted with hiPSC-derived RPE cells differentiated on LN521-coated dishes (i.e. hiPSC-derived RPE cells differentiated on xeno-free biomaterial) were a significantly greater than those of RCS rats injected with PBS solution (negative control) at spatial frequencies of 0.1 and 0.15 cycles/degree 3 weeks after cell transplantation (Figure 8G(i)). The rats became too large after growing for 4 weeks; this led to unsuitability for analysis of optomotor responses using the available qOMR instrument and led to excessive variation in the qOMR results (Figure 8H(ii)).

**Figure 9.**
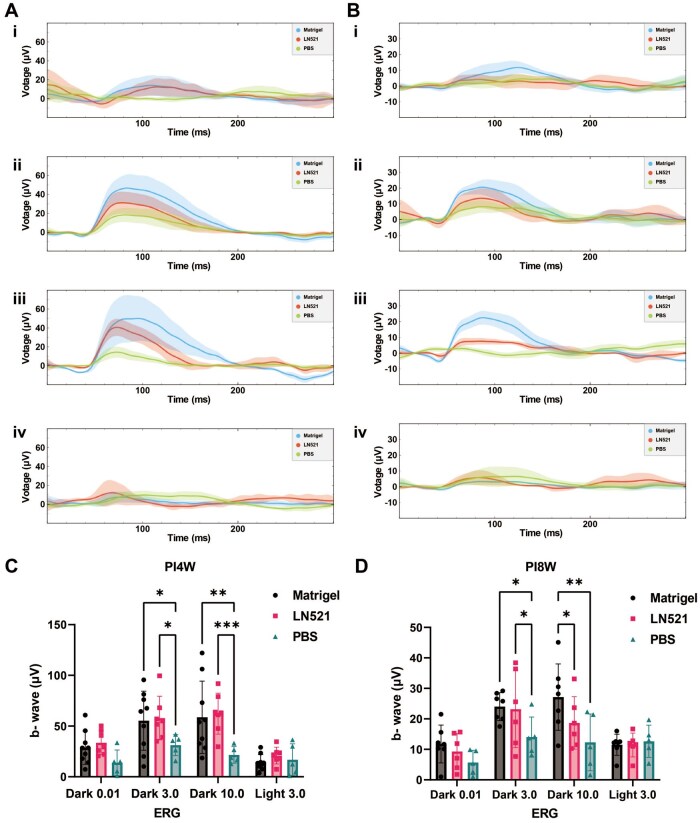
ERG eye responses after cell transplantation (for which the cells were differentiated on Matrigel-coated and LN521-coated dishes) or PBS injection. (**A**) Representative photopic ERG waveform from RCS rats at 4 weeks after cell transplantation or PBS injection. Dark-adapted ERGs at the dark 0.01 (i), dark 3.0 (ii), dark 10.0 (iii) and light 3.0 (iv) cd s/m^2^ intensities. (**B**) Representative photopic ERG waveform from RCS rats at 8 weeks after cell transplantation or PBS injection. Dark-adapted ERGs at 0.01 (i), 3.0 (ii), 10.0 (iii) and light. 10.0 (iv) cd s/m^2^ intensities. (**C**) The average amplitudes of scotopic *b*-waves at dark 0.01, dark 3.0, dark 10.0, and light 3.0 cd·s/m^2^ intensities in RCS rats at 4 weeks after cell transplantation (differentiated on Matrigel-coated (*n* = 9) and LN521-coated (*n* = 7) dishes) or PBS injection (*n* = 5)). (**D**) The average amplitudes of scotopic *b*-waves at dark 0.01, dark 3.0, dark 10.0 and light 3.0 cd·s/m^2^ intensities in RCS rats at 8 weeks after cell transplantation (differentiated on Matrigel-coated (*n* = 8) and LN521-coated (*n* = 6) dishes) or PBS injection (*n* = 5)). The data are presented as the mean ± SDs. *P <* *0.05, **P <* *0.01, ***P <* *0.001.

A decrease in the amplitude of the b-wave and a latency of the peak time of the b-wave are indicative of early death of rod photoreceptor cells [69], and the results of the ERG assay of RCS rats after transplantation of hiPSC-derived RPE cells that were differentiated on Matrigel-coated and LN521-coated dishes are shown in Figure 9. The analysis of the ERG data was conducted based on a previous report [70]. Briefly, we initially implemented principal component analysis (PCA) for noise reduction within the electroretinography (ERG) data, focusing on the first 6 principal components. Subsequently, a third-order polynomial was utilized to approximate the baseline for each individual trace. The derived trend was then subtracted from the trace to achieve baseline correction. Upon the completion of the baseline adjustment, we applied a one-class support vector machine (SVM) algorithm to each trace within the experimental group. The purpose of this classification was to distinguish between ‘normal’ and ‘abnormal’ traces, with the latter being identified as outliers for exclusion from further analysis. The hyperparameter nu (*ν*) was set at 20%, indicating the proportion of outliers expected in the dataset, and the kernel coefficient was fixed at 0.02 to optimize the classification process (Figure 9A and B). The solid lines represent the mean values, which visually show that the RCS rats transplanted with hiPSC-derived RPE cells differentiated on Matrigel-coated or LN521-coated dishes (blue and red lines), had a greater b-wave amplitude than controls transplanted with PBS (green lines, negative control) at Dark 3.0 and 10.0 cd/m^2^ (flash intensity) four weeks (Figure 9A(ii) and (iii)) or eight weeks (Figure 9B(ii) and (iii)) after cell transplantation. Moreover, the statistical analysis of the b-wave of RCS rats transplanted with hiPSC-derived RPE cells revealed stronger ERG responses at Dark 3.0 cd/m^2^ or Dark 10.0 cd/m^2^ than did those of control RCS rats transplanted with PBS (negative control) at 4 weeks (Figure 9C) or 8 weeks (Figure 9D) after cell transplantation, suggesting that the transplantation of hiPSC-derived RPE cells differentiated on Matrigel-coated and LN521-coated dishes, can preserve the visual function of an RCS rat model of retinal degeneration.

## Discussion

Two modified protocols (a modified NIC84 protocol [30] and an activin A [32] protocol) and four cell culture biomaterials (Matrigel, rVN, LN511 and LN521) were evaluated for their ability to induce RPE cell differentiation from hPSCs. The number of cells and the expression of RPE-related markers such as PAX6 (neural marker), MITF (small eye transcription factor), RPE65 (mature RPE marker), and ZO1 (tight junction protein) [71] were calculated as indicators to explore the efficiency of RPE differentiation from hPSCs.

The activin A protocol was not feasible for producing many hPSC-derived mature RPE cells cultured on ECM protein-coated dishes except for Matrigel-coated dishes. However, a 67% increase in the expression of RPE65 was detected by flow cytometry on hiPSC-derived RPE cells differentiated on Matrigel-coated dishes using the activin A protocol, even on Day 28, compared to hiPSC-derived RPE cells differentiated on any of the ECM protein-coated dishes using the modified NIC84 protocol. We replaced Matrigel-coated dishes with dishes coated with mixed ECM protein solutions, such as a mixed solution of rVN, LN511 and LN521; we speculated that these cell culture materials would lead to efficient differentiation of hPSCs into mature and pure RPE cells via the activin A protocol under xeno-free conditions.

According to the modified NIC84 protocol, CTM plays an extremely important role in the directed differentiation of hPSCs into RPE cells. However, a high CTM concentration often leads to cell death during the differentiation process [72]. Therefore, we decided to increase the CTM concentration stepwise over 3-day intervals from 0 to 10, 20 and 30 nM by changing the culture medium. PAX6 expression was detected using a modified NIC84 protocol from Day 28 to Day 84. Notably, the cells that differentiated from Day 56 until Day 84 still expressed PAX6, although PAX6 is an early marker of RPE. This finding has also been reported in several previous articles [73, 74]. These articles mentioned that the PAX6 marker could be detected when pigmentation of the cells became first visible, which indicated that mature RPE cells could also express PAX6 [73, 74]. Furthermore, ZO-1 was expressed continuously throughout the differentiation process, suggesting that the cells maintained their cell–cell junctions and RPE-like characteristics. Different ECM proteins have different affinities for hPSCs and their differentiated RPE cells because ECM proteins bind to different integrin receptors on hPSCs depending on the ECM proteins. However, RPE65 expression was consistently greater than 70% in differentiated cells cultured on any of the ECM protein-coated dishes. One possible reason is that the differentiation medium RPEMN may be not suitable for the survival of hPSCs that have failed to differentiate into RPE cells. Therefore, the remaining RPE cells exhibit a high expression of RPE65, although ECM proteins contribute to the adhesion of RPE cells to ECM proteins, which leads to the increased production of RPE cells.

The modified NIC84 protocol has the advantages of a simplified method of separation and purification and low technical requirements for establishing hiPSC-derived RPE cells, and it has a wide range of applications. However, mature RPE cell markers are expressed at lower levels in the early stage of this protocol, and more time is needed to expand mature RPE cells by isolation and purification. The addition of activin A to the modified NIC84 protocol to induce the differentiation of hPSCs into RPE cells might also be considered in the future.

**Figure 10.**
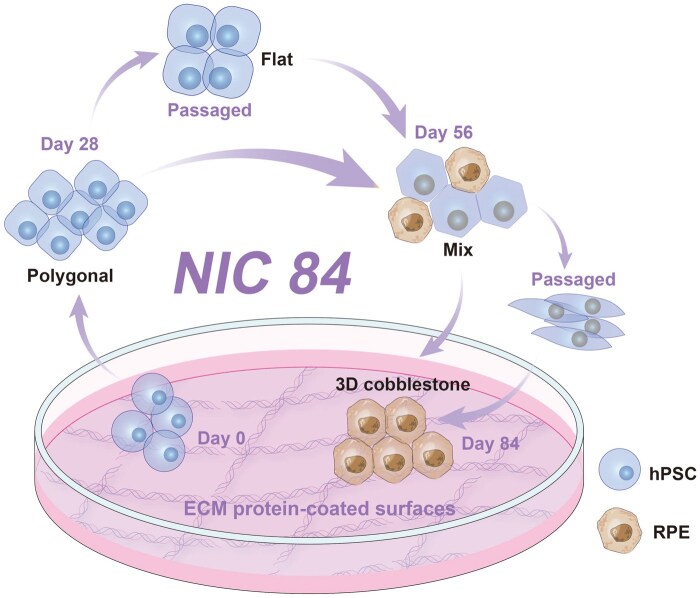
Illustration of the several cell morphological changes that occur during the differentiation of hPSCs into RPE cells following the modified NIC84 protocol.

Some distinct phenomena were observed during the differentiation of hPSCs into RPE cells using the modified NIC84 protocol. First, the differentiated cells exhibited polygonal structures with little difference from Day 7 to Day 28. Second, the boundaries of the cells gradually disappeared after passage on Day 28, and the cells merged to form flat sections within 3 days. Subsequently, small parts of the RPE structure started to appear among these flat areas approximately one week after passage (near Day 40). Finally, the mature RPE cells dispersed evenly in the dishes, while their structures were lost after the cells were passaged on Day 56. Subsequently, the morphology of the mature RPE cells was restored in the following days (Figure 10). Some studies have reported that a similar phenomenon may be epithelial–mesenchymal transition (EMT) [75, 76], which needs to be further verified in subsequent studies.

Pennington *et al.* [77] investigated the maintenance of hESCs and their differentiation into RPE cells using Synthemax II-SC (a synthetic copolymer compliant with a peptide derived from the rVN sequence). Rowland et al. selected several ECM proteins and substrates and tested their ability to support hPSC-RPE differentiation and maintenance with Matrigel and mouse laminin-111, which support the highest pigmentation frequency [78]. Zhu *et al.* [79] compared the ability of LN521 and rVN to adhere to hESC-RPE cells. Notably, fully differentiated hESC-RPE cells were used in these experiments. In a study by Viheriälä *et al.* [80], type IV collagen (Col-IV), laminin (LN) and nidogen-1 were found to be involved in the maturation and functionality of hESC-RPE cells after cryopreservation.

In addition, the therapeutic effect of hiPSC-derived RPE cells was evaluated in RCS rats, an animal model of retinal degenerative disease [81]. The RCS rat model is a well-established model of retinal degeneration caused by a mutation in the MER proto-oncogene tyrosine kinase (*Mertk*). RPE cells in RCS rats cannot phagocytose the outer segments of photoreceptor cells, which leads to progressive death of photoreceptor cells [62, 82–85]. The apoptosis of photoreceptor cells begins at approximately 3 weeks after birth in RCS rats, and the photoreceptor cells almost always die when the rats are between 8 and 12 weeks of age, which leads to severe loss of the retinal structure and function [62, 82–86]. As in previous studies, progressive retinal degeneration in RCS rats could be rescued by subretinal transplantation of human RPE cells [87], human neural progenitor cells [88], hPSC-derived photoreceptor cells [84] and RPE cells [62, 84], which together with our results further validated the possibility of the use of xenogeneic RPE for the treatment of retinal degenerative diseases.

## Conclusion

The activin A protocol is not preferable for hPSC differentiation into RPE cells cultured on xeno-free ECM protein-coated dishes. It is more efficient to generate hPSC-derived RPE cells using the modified NIC84 protocol than the activin A protocol or the original NIC84 protocol. We succeeded in developing an optimized NIC84 protocol for the efficient differentiation of hPSCs into RPE cells cultured on xeno-free LN521-coated dishes although most researchers have used rVN-coated dishes [89–92], which were less promising for the differentiation of hPSCs into RPE cells in this study. Fundus imaging and OCT confirmed the successful transplantation of hiPSC-derived RPE cells into the subretinal spaces of RCS rats. RCS rats subjected to transplantation of hPSC-derived RPE cells differentiated on xeno-free cell culture material (LN521-coated dishes), exhibited better visual function than did those in the sham control group after transplantation, as determined via qOMR and ERG.

## Supplementary Material

rbae091_Supplementary_Data

## Data Availability

The data that support the findings of this study are available from the corresponding author upon reasonable request.
