# Supplementary material for: Effects of ECM protein-coated surfaces on the generation of retinal pigment epithelium cells differentiated from human pluripotent stem cells
Source: Regen Biomater. 2024 Aug 20;11:rbae091. doi: 10.1093/rb/rbae091 (PMC11374035; doi:10.1093/rb/rbae091)
Supplement: rbae091_Supplementary_Data [file rbae091_supplementary_data.pdf]

Effects of ECM protein-coated surfaces on the generation of retinal pigment epithelium cells differentiated from human pluripotent stem cells

Zeyu Tian[1,†], Qian Liu[1,†], Hui-Yu Lin[2], Yu-Ru Zhu[2], Ling Ling[1], Tzu-Cheng Sung[1], Ting Wang[1], Min Gao[1], Wanqi Li[1], Sitian Cheng[1], Remya Rajan Renuk[3], Suresh Kumar Subbiah[3], Guoping Fan[4], Gwo-Jang Wu[5], Akon Higuchi[1,2,6*]

[1] State Key Laboratory of Ophthalmology, Optometry and Visual Science, Eye Hospital, Wenzhou Medical University, No. 270, Xueyuan Road, Wenzhou, 325027, Zhejiang, China.

[2] Department of Chemical and Materials Engineering, National Central University, No. 300, Jhongda RD., Jhongli, 32001, Taoyuan

[3] Center for Global Health Research, Saveetha Medical College and Hospitals, Saveetha Institute of Medical and Technical Sciences, Chennai, 602105, Tamil Nadu, India.

[4]Department of Human Genetics, David Geffen School of Medicine, UCLA, Los Angeles, CA 90095, USA.

[5]Graduate Institute of Medical Sciences and Department of Obstetrics & Gynecology, Tri-Service General Hospital, National Defense Medical Center, Taipei, 11490, Taiwan

[6]R&D Center for Membrane Technology, Chung Yuan Christian University, Chungli, Taoyuan 320, Taiwan

* Correspondence and requests for materials should be addressed to A.H. (e-mail: higuchi@ncu.edu.tw & higuchi@wmu.edu.cn)

[†] These authors contributed equally.

List of Supplementary Materials

Table S1. Materials used in this study.

Table S2. Antibodies used in this study.

Supplementary Figures

Fig. S1. Generation of hiPSC-derived RPE cells using the Activin A protocol.

Fig. S2. Expression of RPE cell markers of hiPSC-RPE cells using flow cytometry at day 28 following the Activin A protocol

Fig. S3. Pigmented cells on Matrigel- and LN521- coated dishes after 84 days of hiPSC differentiation following the modified NIC84 protocol.

Fig. S4. Differentiation of hESCs (H9) and hiPSCs (Mix-2) into RPE cells using modified NIC84 protocol.

Fig. S5-S6. Expression of RPE cell markers of hiPSC-derived RPE cells using immunostaining at day 28 and day 56 following the modified NIC84 protocol.

Fig. S7-S9. Expression of RPE cell markers of hiPSC-derived RPE cells using flow cytometry at day 28, day 56 and day 84 following the modified NIC84 protocol.

Supplementary Information

Supplementary Table 1 Materials used in this study.

Materials	Abbreviation	Catalog No.	Company
		**ECM**	
Matrigel	MAT	356230	Corning, Bedford, USA
Recombinant vitronectin	rVN	A14700	Gibco, Frederick, USA
Biolaminin 511	LN511	LN511-0202	BioLamina, Sundbyberg, Sweden
Biolaminin 521	LN521	LN521-02	BioLamina, Sundbyberg, Sweden
		**Activin A protocol**	
Recombinant Activin A	Activin A	120-14E	PeproTech, London, UK
DMEM/F12, GultaMAX medium	DMEM/F12	10565018	Gibco, Grand Island, USA
Neurobasal medium	NM	21103049	Gibco, Grand Island, USA
N2 supplement	N2	17502048	Gibco, Grand Island, USA
B27 supplement	B27	17504044	Gibco, Grand Island, USA
DMEM, high glucose medium	DMEM, high glucose	11965092	Gibco, Grand Island, USA
L-glutamine supplement	L-glutamine	25030081	Gibco, Grand Island, USA
2-Mercaptoethanol	2-Mercaptoethanol	21985023	Gibco, Grand Island, USA
MEM Non-essential Amino Acid Solution	MEM NEAA	11140050	Gibco, Scotland, UK
KnockOut Serum Replacement	KOSR	10828010	Gibco, Grand Island, USA
Penicillin-streptomycin	PS	15070063	Gibco, Grand Island, USA
		**NIC84 protocol**	
Nicotinamide	NIC	N0636	Sigma, Darmstadt, Germany
Chetomin	CTM	C9623	Sigma, Darmstadt, Germany
DMEM medium	DMEM	11056016	Gibco, Grand Island, USA
DMEM/F-12 medium	DMEM/F-12	11320033	Gibco, Grand Island, USA
Ham's F-12 medium	F-12	11765054	Gibco, Grand Island, USA
GlutaMAX Supplement	GlutaMAX	35050061	Gibco, Grand Island, USA
Antibiotic-Antimycotic	Anti-Anti	15240062	Gibco, Grand Island, USA
		**Cell culture medium and component**	
mTeSR1 medium	mTeSR1	85850	STEMCELL, Vancouver, Canada
Accutase solution	Accutase	A6964	Sigma, Darmstadt, Germany
Dispase II	Dispase	D4693	Sigma, Darmstadt, Germany
Rock inhibitor	Y-27632	72302	STEMCELL, Vancouver, Canada
Dimethyl sulfoxide	DMSO	D8371	Solarbio, Beijing, China
		**Other materials**	
DPBS, Calcium, Magnesium	DPBS, Ca^{2+}, Mg^{2+}	14040133	Gibco, Grand Island, USA
DPBS, no Calcium, no Magnesium	DPBS	14190144	Gibco, Grand Island, USA
4% paraformaldehyde	4% PFA	P0099	Beyotime, Shanghai, China
Permeabilization Buffer with Triton X-100	Triton X-100	P0096	Beyotime, Shanghai, China
QuickBlock Blocking Buffer	Blocking buffer	P0102	Beyotime, Shanghai, China
Antibody Dilution Buffer	Dilution Buffer	P0103	Beyotime, Shanghai, China
Wash Buffer	Wash Buffer	P0106	Beyotime, Shanghai, China
CellTracker Green CMFDA Dye	CellTracker Green	C2925	Invitrogen, Eugene, USA

Supplementary Table 2 Antibodies used in this study.

Name	Catalog No.	Company	Concentration	Dilution rate
ZO1 Rabbit Antibody	40-2200	Invitrogen, Eugene, USA	0.25mg/ml	Immunostaining (1:50-1:100), Flow cyto. (1:50-1:100)
PAX6 Mouse Antibody	13B10-1A10	Invitrogen, Eugene, USA	1mg/ml	Immunostaining (1:50-1:200), Flow cyto.（1:100）
MITF Rabbit Antibody	MA5-32554	Invitrogen, Eugene, USA	1mg/ml	Immunostaining (1:50-1:100), Flow cyto. (1:50-1:100)
RPE65 Mouse Antibody	MA1-16578	Invitrogen, Eugene, USA	1mg/ml	Immunostaining (1:50-1:200), Flow cyto. (1:100)
Mouse IgG Isotype Control	MA5-14453	Invitrogen, Eugene, USA	0.1mg/ml	Flow cyto. (1:100)
Rabbit IgG Isotype Control	10500C	Invitrogen, Eugene, USA	3mg/ml	Assay-Dependent
Goat Anti-Rabbit IgG H&L (Alexa Fluor 488)	ab150077	Abcam, Carlsbad, USA	2mg/ml	Immunostaining (1:200-1:1000), Flow cyto. (1:2000-1:4000)
Goat Anti-Mouse IgG H&L (Alexa Fluor 555)	ab150114	Abcam, Carlsbad, USA	2mg/ml	Immunostaining (1:200-1:2000), Flow cyto. (1:2000)
Goat Anti-Rabbit IgG H&L (PE)	ab72465	Abcam, Carlsbad, USA	0.1mg/ml	Flow cyto. (1:500-1:1000)
Goat Anti-Mouse IgG H&L (FITC)	ab6785	Abcam, Carlsbad, USA	Affinity purified	Flow cyto. (1:500-1:2500)
DAPI	C1006	Beyotime, Shanghai, China	-	-

Supplementary Figures

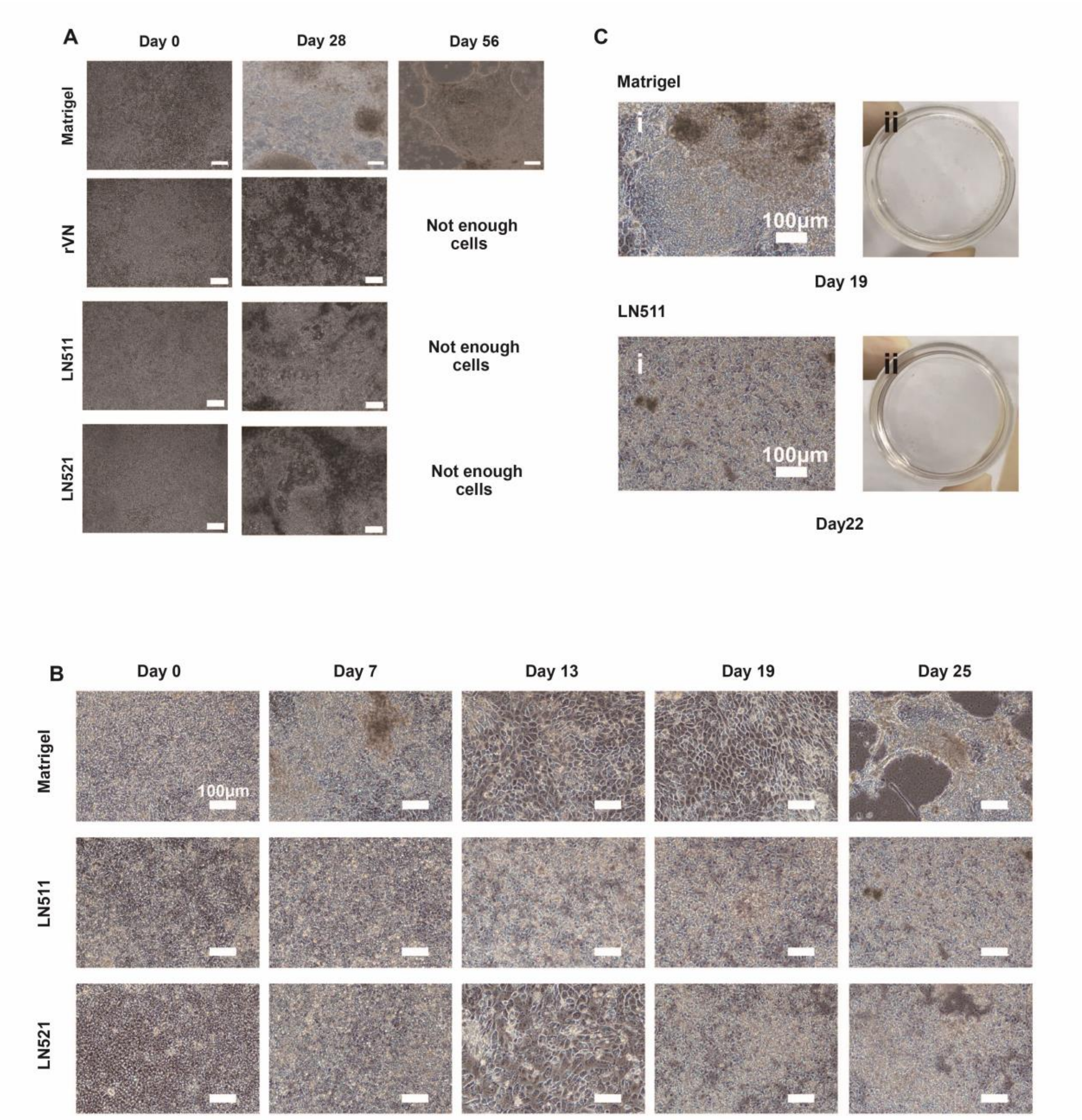

Supplementary Fig. 1 Generation of hiPSC-derived RPE cells using the Activin A protocol. **A.** Morphology of hiPSC-RPE cells following the original Activin A protocol (50 ng/mL Activin A). Scale bar: 100 μm. **B.** Morphology of hiPSC-RPE cells following the Activin A protocol (25 ng/mL Activin A). Scale bar: 100 μm. **C.** Pigmented cells can be observed on Matrigel-coated dishes following the Activin A protocol (25 ng/mL Activin A) at day 19 or on LN511-coated dishes at day 22. Scale bar: 100 μm.

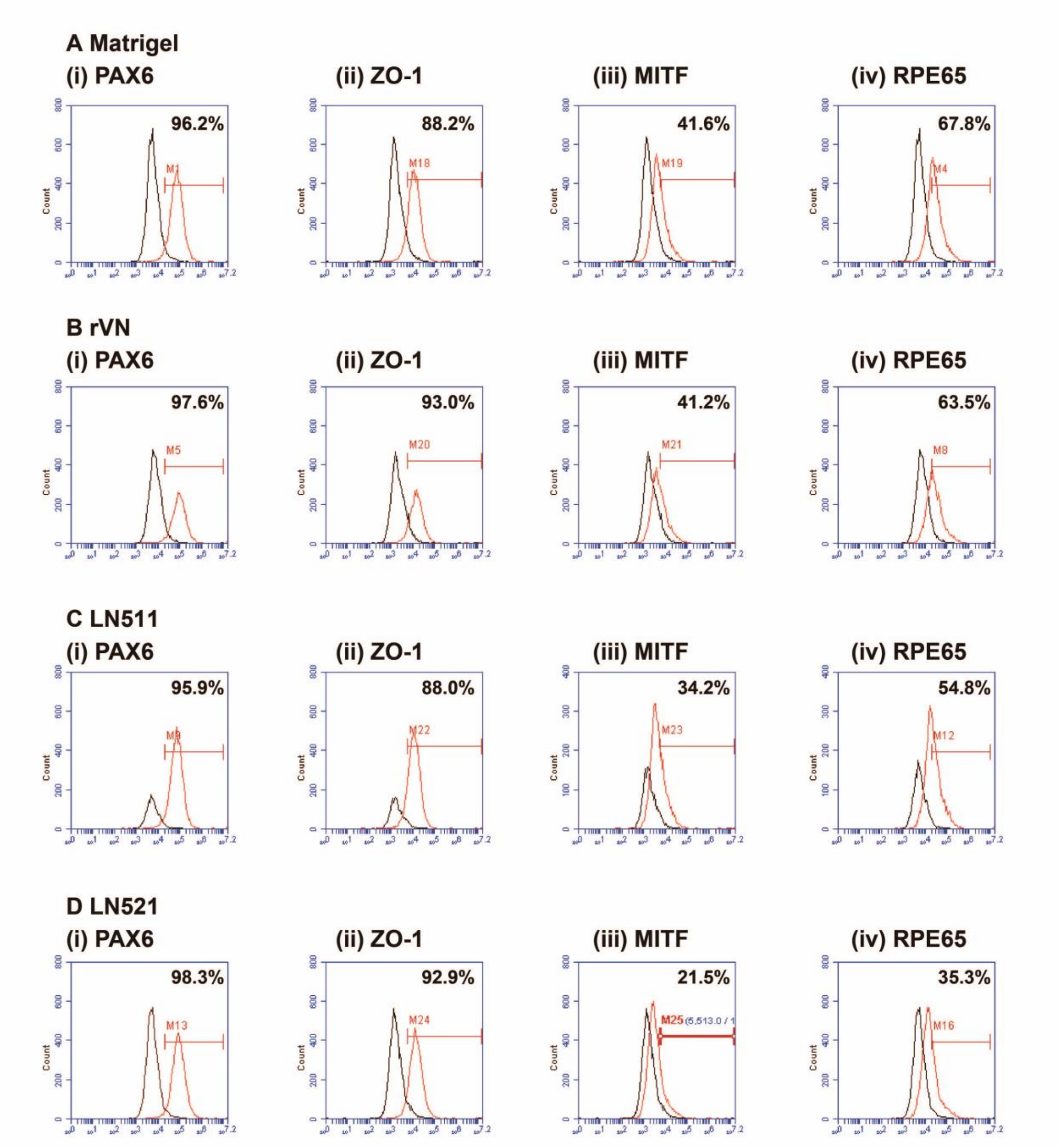

Supplementary Fig. 2 Expression of RPE cell markers (PAX6 (i), ZO-1(ii), MITF(iii), RPE65(iv)) of hiPSC-RPE cells cultured on Matrigel-**(A)**, rVN-**(B)**, LN511-**(C)**, and LN521-**(D)** coated dishes using flow cytometry at day 28 following the Activin A protocol (50 ng/mL Activin A). The black line represents the cells stained with isotype antibody.

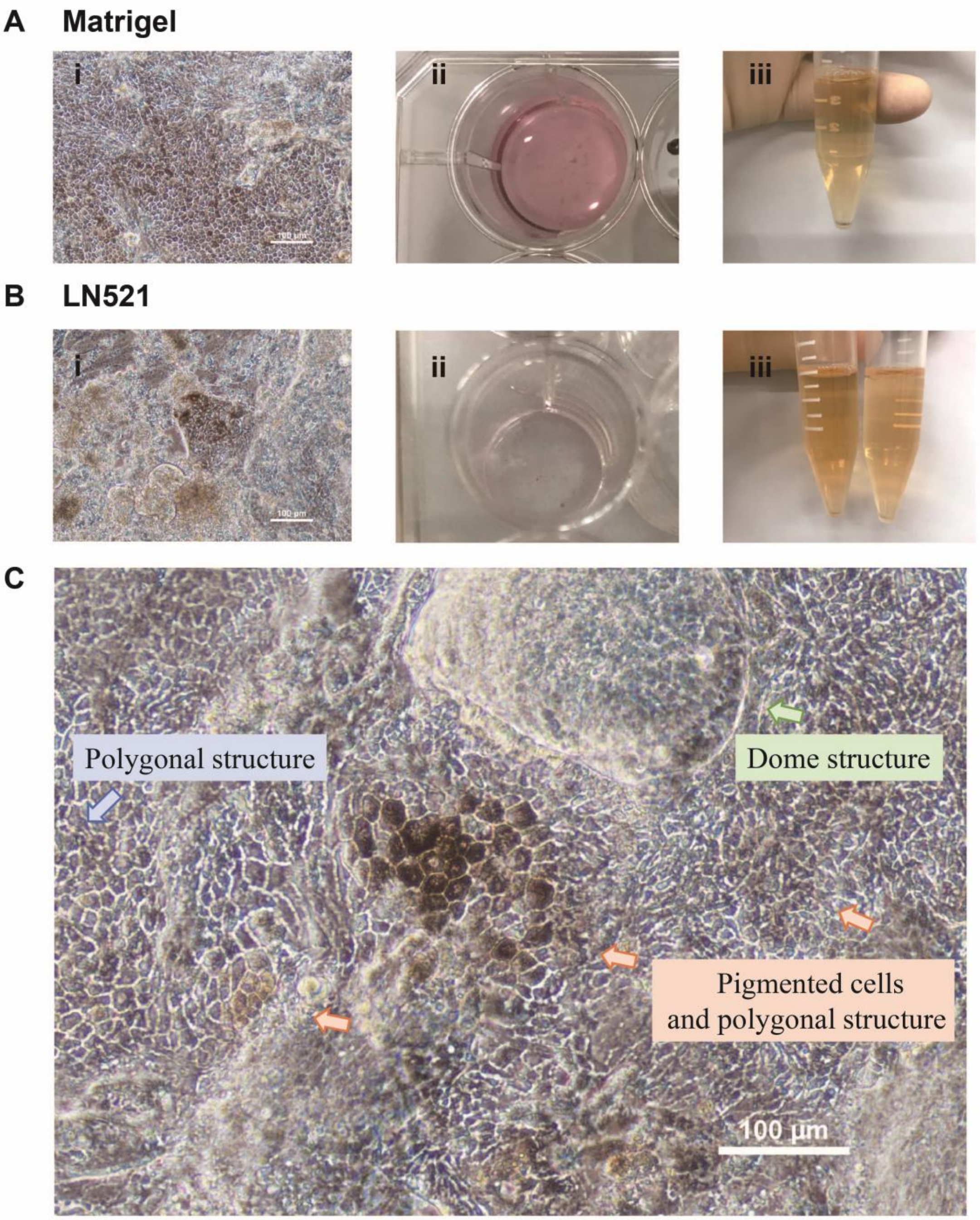

Supplementary Fig. 3 Pigmented cells can be observed on Matrigel-(**A**) and LN521-(**B**) coated dishes after 84 days of hiPSC differentiation following the modified NIC84 protocol. Scale bar: 100 µm. **C.** hiPSC-derived RPE cells with different levels of maturity appeared on Matrigel-coated dish following the modified NIC84 protocol. Scale bar: 100 µm.

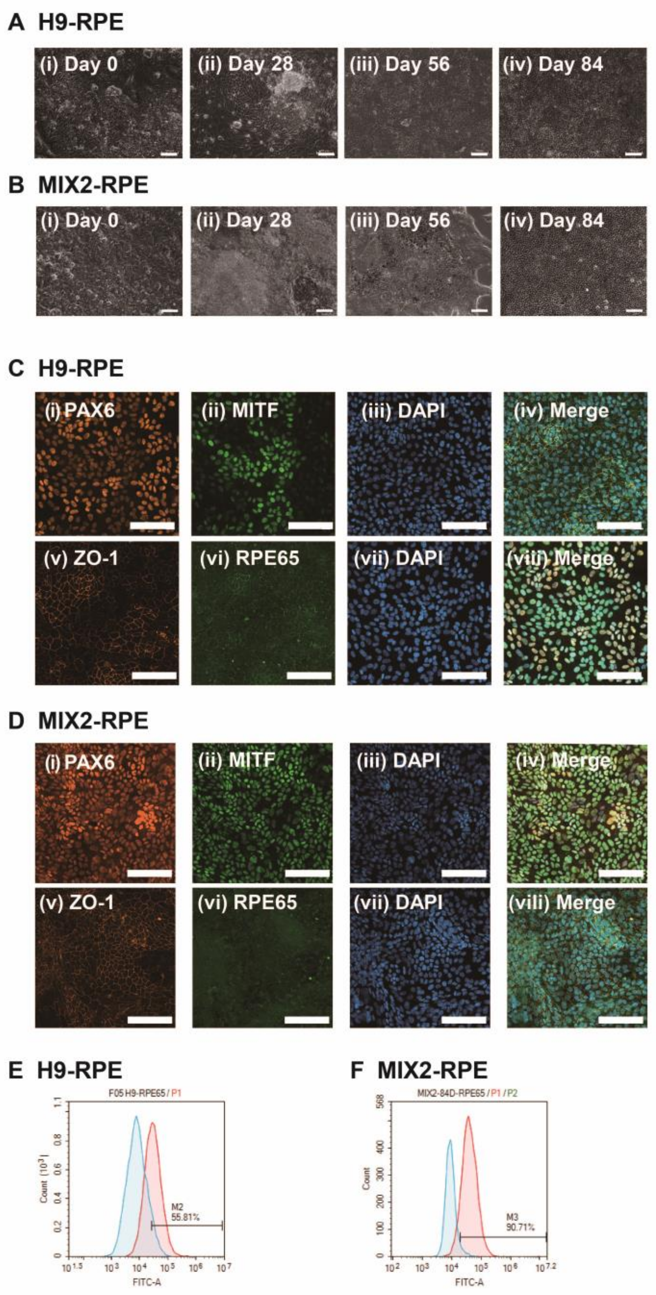

Supplementary Fig. 4 Differentiation of hESCs (H9) and hiPSCs (Mix-2) into RPE cells using modified NIC84 protocol. **A** The cell morphology of RPE cells derived from hESCs using the modified NIC84 protocol cultured on Matrigel-coated dishes at day 0 (i), day 28 (ii), day 56 (iii), and day 84 (iv). Scale bar: 50 μm. **B** The cell morphology of RPE cells derived from hiPSCs (Mix-2) cultured on Matrigel-coated dishes at day 0 (i), day 28 (ii), day 56 (iii), and day 84 (iv). Scale bar: 50 μm. **C** Expression of RPE cell markers (PAX6, ZO-1, MITF, RPE65) of hESC(H9)-RPE cells cultured on Matrigel-coated dishes using immunostaining at day 84. Nuclei were stained with DAPI (iii, vii). The photos in (iv) and (viii) were created by merging (i) - (iii) and (v) - (vii), respectively. Scale bar: 100 μm. **D** Expression of RPE cell markers (PAX6, ZO-1, MITF, RPE65) of hiPSC(Mix2)-RPE cells cultured on Matrigel-coated dishes using immunostaining at day 84 following the modified NIC84 protocol. Nuclei were stained with DAPI (iii, vii). The photos in (iv) and (viii) were created by merging (i) - (iii) and (v) - (vii), respectively. Scale bar: 100 μm. **E** Expression of RPE65 of hESC(H9)-RPE cells cultured on Matrigel-coated dishes using flow cytometry at day 84. The blue line represents the the cells stained with isotype antibody. **F** Expression of RPE65 of hiPSC(Mix2)-RPE cells cultured on Matrigel-coated dishes using flow cytometry at day 84. The blue line represents the cells stained with isotype antibody.

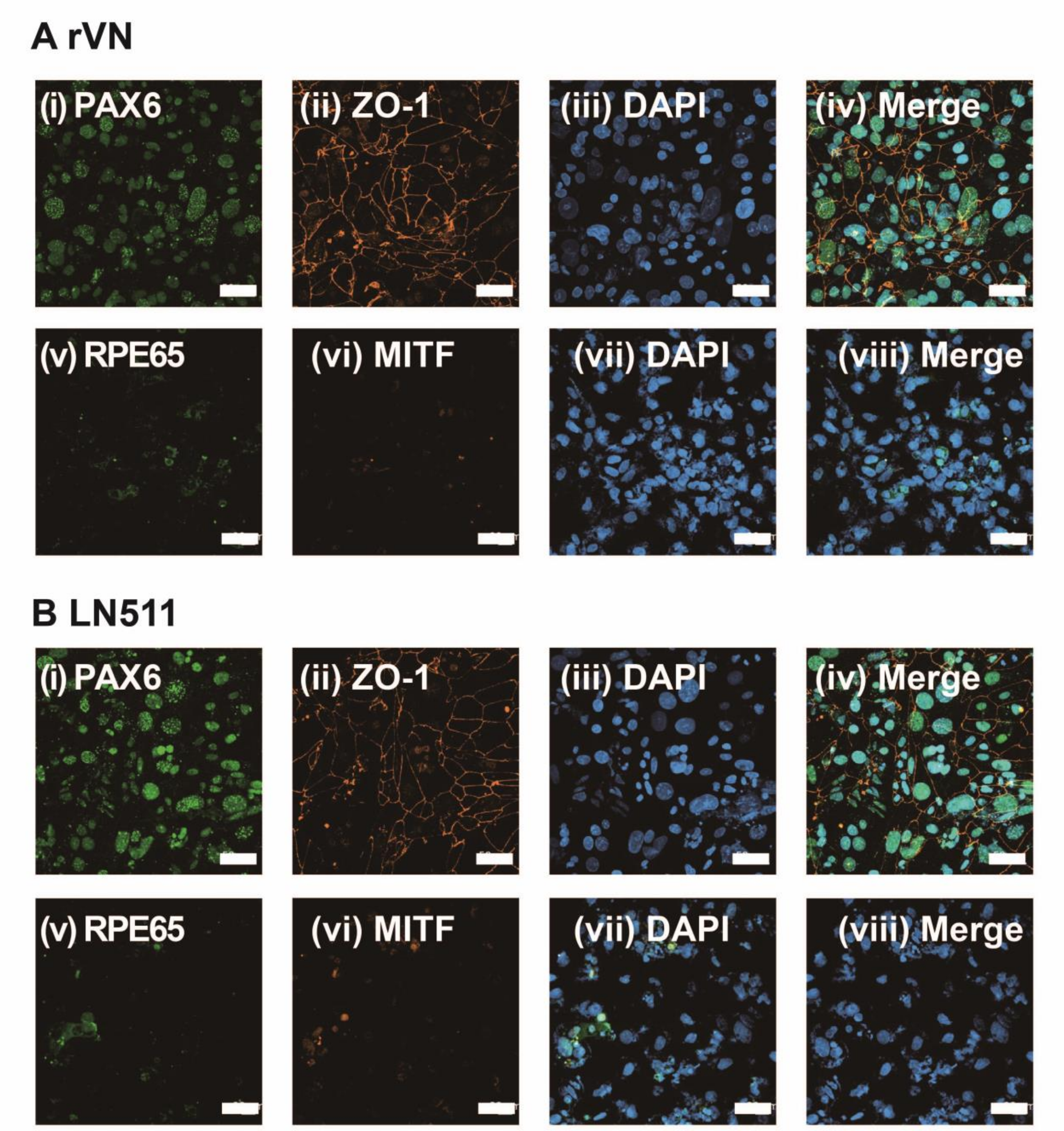

Supplementary Fig. 5 Expression of RPE cell markers (PAX6 (i), ZO-1 (ii), RPE65 (v), MITF (vi)) of hiPSC-derived RPE cells cultured on rVN-(**A**) and LN511-(**B**) coated dishes using immunostaining at day 28 following the modified NIC84 protocol. Nuclei were stained with DAPI (iii, vii). The photos in (iv) and (viii) were created by merging (i) - (iii) and (v) - (vii), respectively. Scale bar: 50 µm.

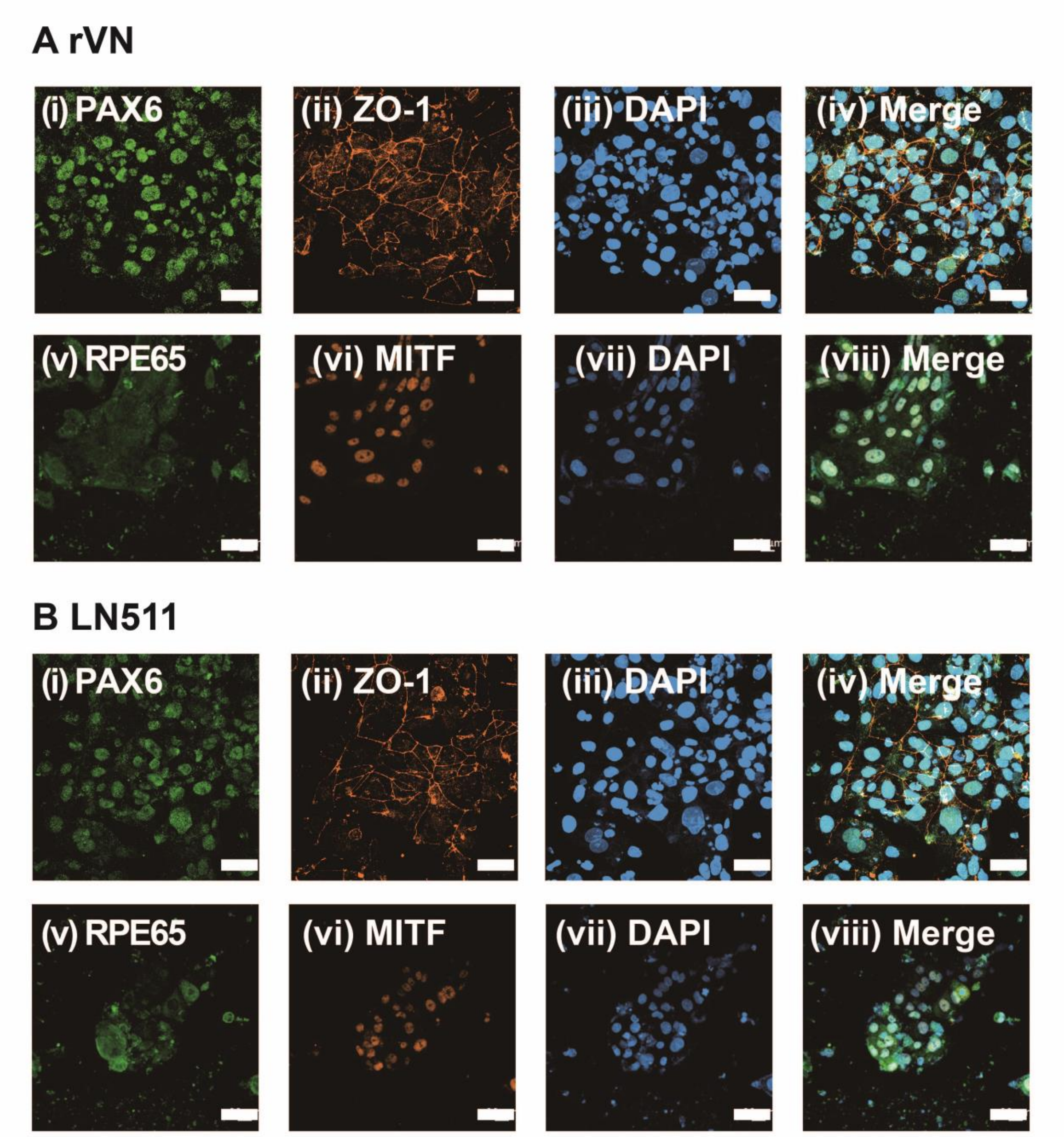

Supplementary Fig. 6 Expression of RPE markers (PAX6 (i), ZO-1 (ii), RPE65 (v), MITF (vi)) of hiPSC-derived RPE cells cultured on rVN-(**A**) and LN511-(**B**) coated dishes using immunostaining at day 56 following the modified NIC84 protocol. Nuclei were stained with DAPI (iii, vii). The photos in (iv) and (viii) were created by merging (i) - (iii) and (v) - (vii), respectively. Scale bar: 50 μm.

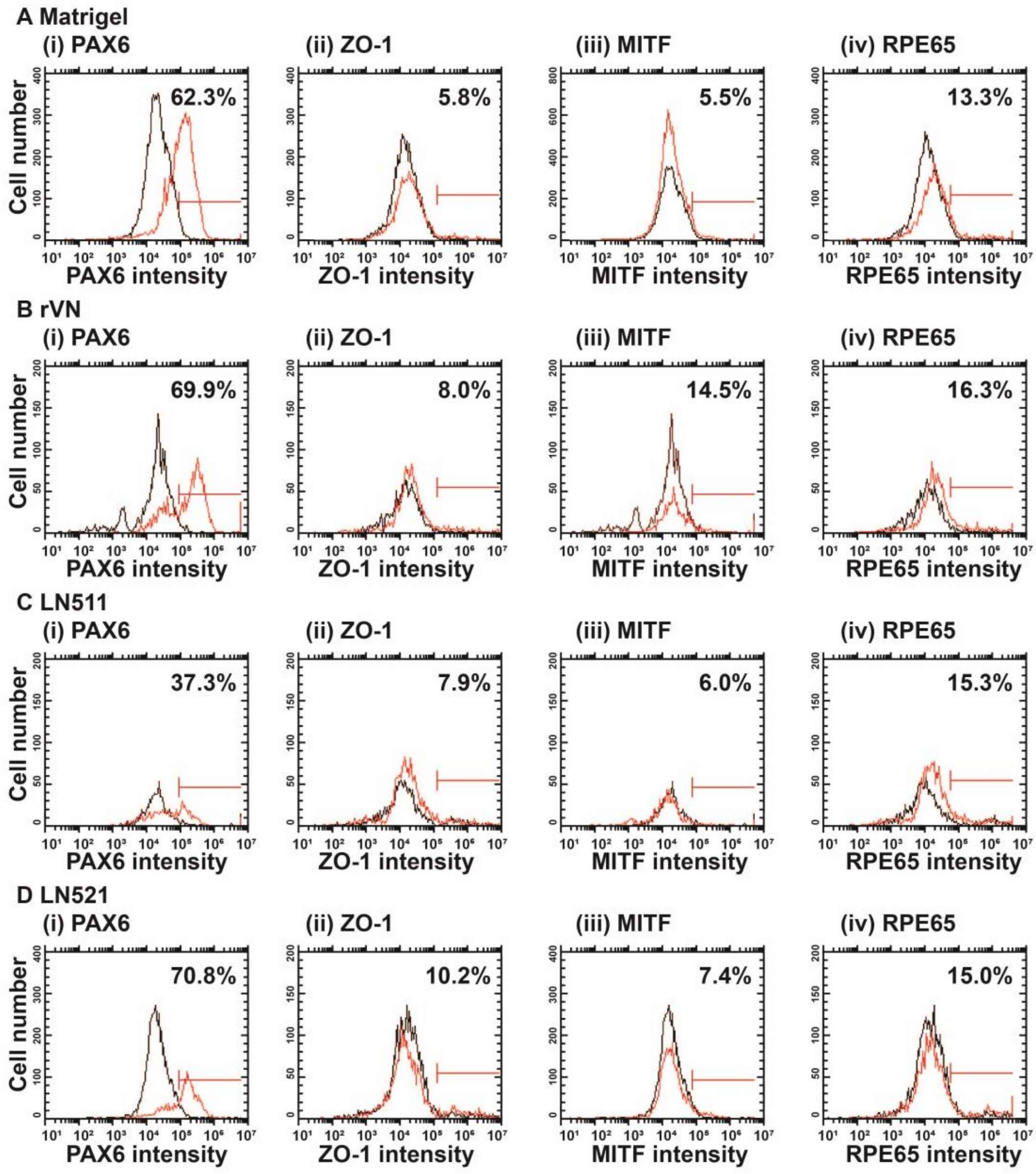

Supplementary Fig. 7 Expression of RPE cell markers (PAX6 (i), ZO-1 (ii), MITF (iii), RPE65 (iv)) of hiPSC-derived RPE cells cultured on Matrigel-(**A**), rVN-(**B**), LN511-(**C**), and LN521-(**D**) coated dishes using flow cytometry at day 28 following the modified NIC84 protocol. The black line represents the cells stained with isotype antibody.

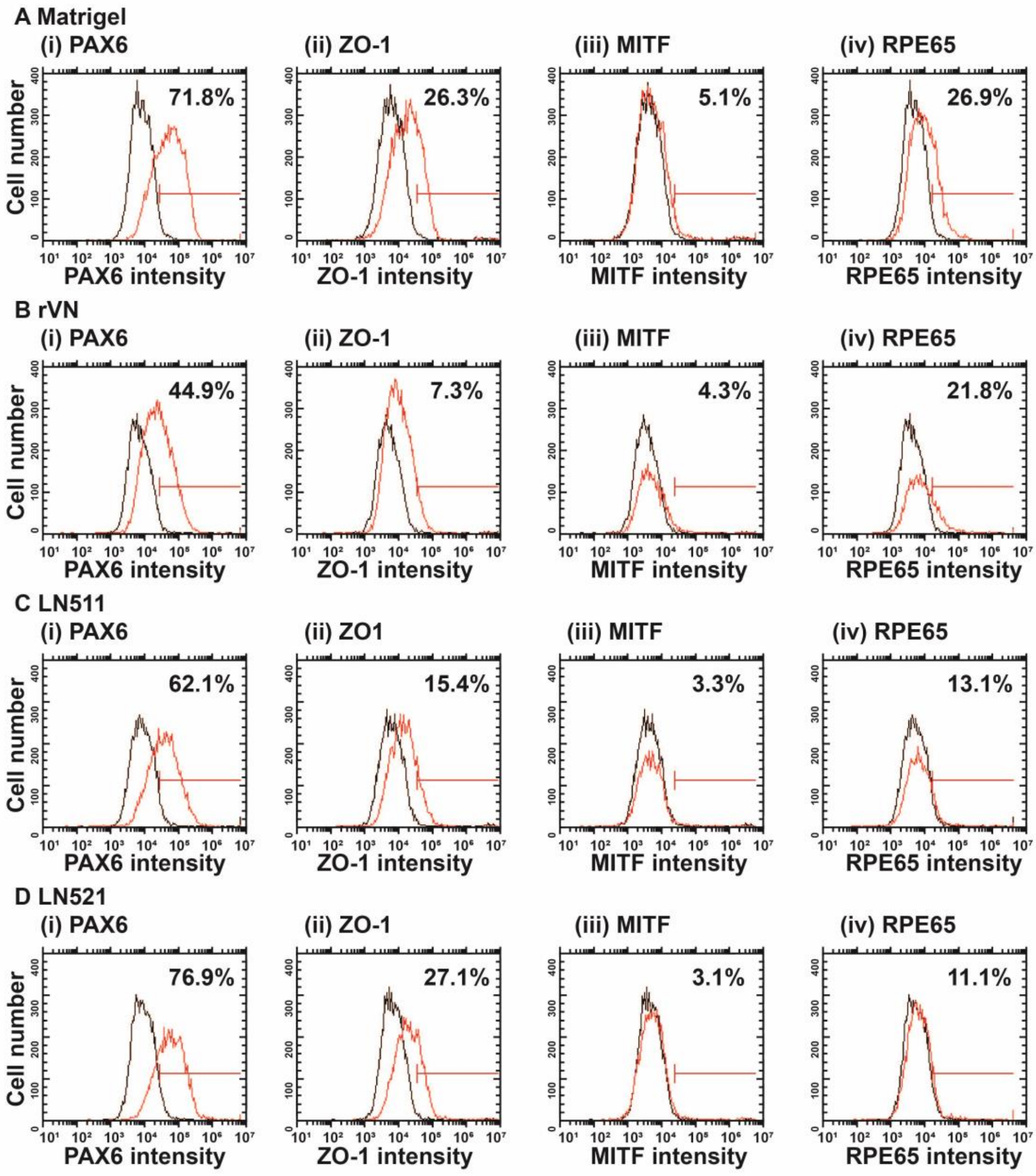

Supplementary Fig. 8 Expression of RPE cell markers (PAX6 (i), ZO-1 (ii), MITF (iii), RPE65 (iv)) of hiPSC-derived RPE cells cultured on Matrigel-(**A**), rVN-(**B**), LN511-(**C**), and LN521-(**D**) coated dishe**s** using flow cytometry at day 56 following the modified NIC84 protocol. The black line represents the cells stained with isotype antibody.

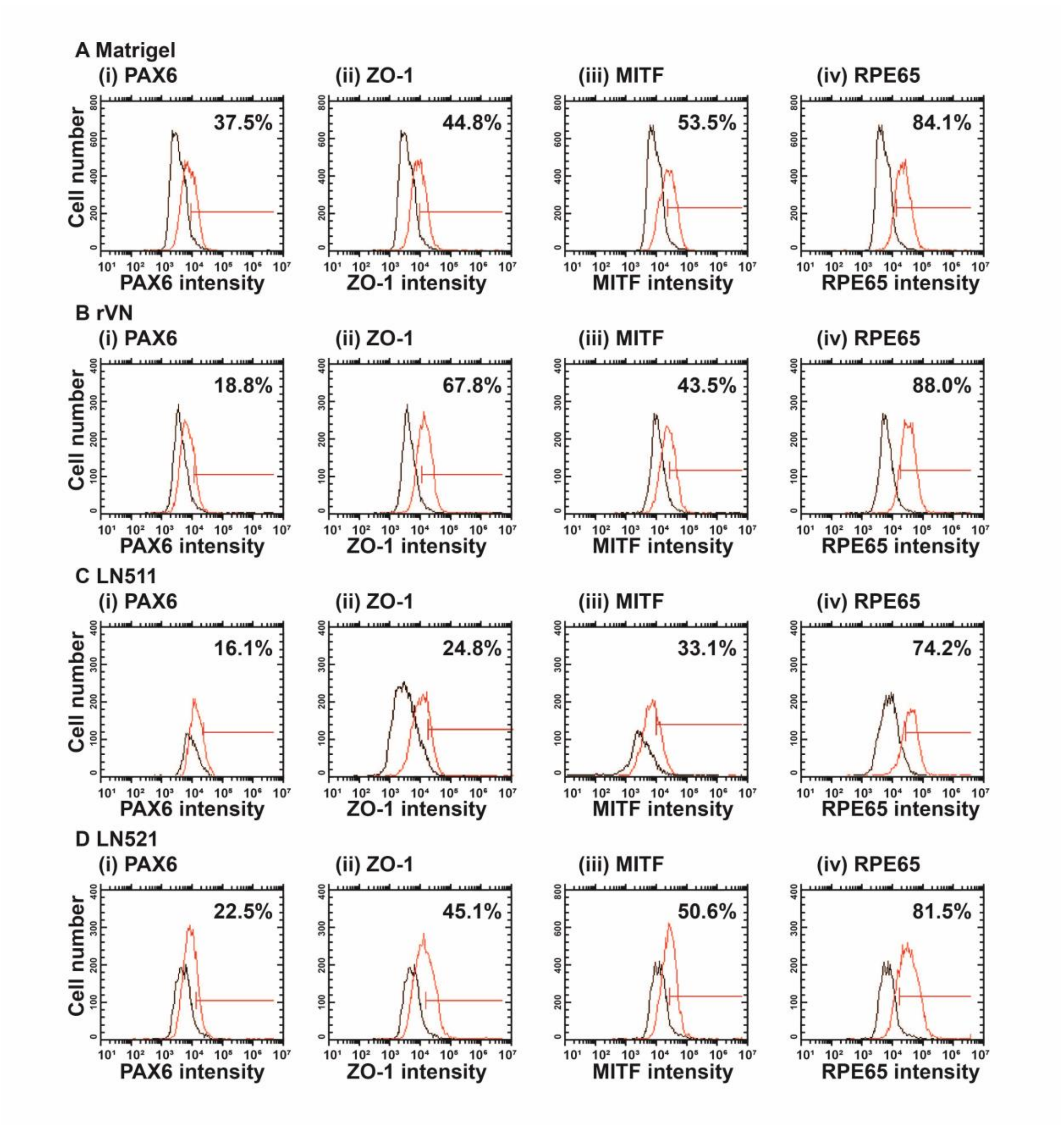

Supplementary Fig. 9 Expression of RPE cell markers (PAX6 (i), ZO-1 (ii), MITF (iii), RPE65 (iv)) of hiPSC-derived RPE cells cultured on Matrigel-(**A**), rVN-(**B**), LN511-(**C**), and LN521-(**D**) coated dishes using flow cytometry at day 84 following the modified NIC84 protocol. The black line represents the cells stained with isotype antibody.
